# The Nutritional Intervention of Resveratrol Can Effectively Alleviate the Intestinal Inflammation Associated With Celiac Disease Induced by Wheat Gluten

**DOI:** 10.3389/fimmu.2022.878186

**Published:** 2022-04-05

**Authors:** Tian Yu, Yiting Xie, Juanli Yuan, Jinyan Gao, Zhiwen Xiao, Yong Wu, Hongbing Chen

**Affiliations:** ^1^ State Key Laboratory of Food Science and Technology, Nanchang University, Nanchang, China; ^2^ Sino-German Joint Research Institute, Nanchang University, Nanchang, China; ^3^ School of Food Science and Technology, Nanchang University, Nanchang, China; ^4^ School of Pharmaceutical Science, Nanchang University, Nanchang, China

**Keywords:** celiac disease, gliadin, intestinal inflammation, oxidative damage, resveratrol, nutritional intervention

## Abstract

**Background and Aims:**

Wheat gluten is a critical trigger for celiac disease, often causing inflammatory lesions and oxidative stress damage in the intestines of patients. In daily life, it is difficult for celiac disease patients to strictly avoid the dietary intake of gluten, which makes complementary preventive therapy particularly urgent. As such, we investigated the alleviating effects of resveratrol *in vivo* and *in vitro* models of celiac disease.

**Methods:**

We established *in vivo* and *in vitro* models of gluten protein-induced celiac disease. The intervention effect of resveratrol was defined well based on relevant indicators of inflammation, immunity and oxidative stress, and its possible involvement in signaling pathways and genes were also identified.

**Results:**

Resveratrol was effective in reducing intestinal oxidative stress and inflammatory damage induced by wheat gluten in both cell and mouse models for celiac disease. We identified correlations between the genes (Fgf15, Nr0b2, Aire and Ubd) and signaling pathways (PPAR, AMPK and FoxO) in which resveratrol performed critical roles.

**Conclusions:**

Resveratrol contributed to regulate development of autoimmunity through up-regulation of Aire and Ubd genes and promote nutrient absorption in intestine through down-regulation of Fgf15 and Nr0b2 genes, as well as played a role in regulating complex response system of oxidative stress, inflammatory response and immune response in intestine by activating PPAR, AMPK and FoxO signaling pathways, thus effectively alleviating the intestinal symptoms of celiac disease.

## Introduction

Celiac disease (CD) is a complex multifactorial disorder involved genetic and environmental factors (1). CD arises from an autoimmune response in which gluten stimulate immune system T cells of genetically susceptible individuals (individuals with human leukocyte antigen DQ2/DQ8) (2). By the activation of tissue transglutaminase (TG2) in intestinal, different glutamine residues in gliadin immunodominant peptides (such as p57-68 and 33-mer) were deamidated into glutamate, which facilitated the formation of immune stimulating epitopes and produces strong binding affinity with HLA-DQ2/DQ8 class II molecules on antigen-presenting cells (3). These peptides triggered the adaptive immune response mediated by CD4^+^Th1 cells and the innate immune response mediated by intraepithelial lymphocytes, which caused intestinal epithelial inflammatory cell infiltration, villus atrophy and crypt hyperplasia (4, 5).

The molecular mechanism of CD involved in inflammation and oxidative stress caused by increased reactive oxygen species and reduced antioxidant defenses (6). Gliadin not only exerted cytotoxic and immunomodulatory activities in the pathogenesis of CD, but also triggered oxidative stress and induced the release of pro-inflammatory factors (7). Toxic gliadin peptides p31-43 in enterocytes could induce tight junction dysfunction and some cytotoxic effects (8), such as apoptosis (such as thymocytes and Th2 cells) and differentiation of T cell (such as Th1, Th17 and regulatory T cells) (9, 10), most of which were mediated by increased oxidative stress. Luciani (11) found that toxic gliadin peptides p31-43 clustered in lysosomes lead to uncontrolled activation of signal transduction pathways including NF-κB and sustained increase in the levels of reactive oxygen species and reactive nitrogen species radicals.

The most effective way to treat CD was that patients must adhere to a gluten-free diet throughout their lives (12). However, it was difficult that patients have to maintain a gluten-free diet for a long time as gluten-containing foods were one of the main patterns of modern diet (13). It was essential to explore new methods of prevention or nutritional intervention for improving intestinal symptoms of CD. The intestinal inflammation and immune response were often associated with oxidative stress (14), and natural antioxidants were effective in inhibiting the oxidative response of free radicals, thereby alleviating oxidative stress and slowing down the inflammatory response (7). Accordingly, we speculated that the use of natural antioxidants supplements could alleviate CD or will be an important addition to the classical treatment of CD.

Resveratrol (3,4,5-trihydroxy-trans-stilbene) was a natural polyphenol found in a variety of medicinal plants, grapes and red wine (15). It was shown to prevent and treat chronic inflammatory diseases by scavenging free radicals and regulating various enzymes (such as COX and iNOS) involved in the inflammatory response (16). In addition, resveratrol not only inhibited inflammatory gene expression, but also suppresses T cell activation and reduced cytokine production, which had a positive effect on the treatment of autoimmune diseases (17). In our study, we established *in vivo* and *in vitro* models for CD involving oxidative stress response. Subsequently, we used these models to explore the effects of nutritional interventions with resveratrol on celiac disease-related conditions as well as the genes/pathways that may be involved. These findings would provide a new solution for the prevention and treatment of celiac disease.

## Materials and Methods

### Reagents

Resveratrol (98%) was bought from FUJIFILM Wako (Osaka, Japan). The p31-43 peptides (LGQQQPFPPQQPY) were synthesized by Sangon Biotech (Shanghai) Co., Ltd. Extraction and purification of ATI were prepared as described by Zevallos (18). Gliadin from wheat was purchased from Sigma-Aldrich (Sigma, Missouri, USA).

### Cell Model

Caco-2 cells were purchased from American type culture collection (ATCC) Cell Lines and stimulated with the gluten protein-derived peptide p31-43. The solvent of p31-43 was PBS in which the PBS group acted as a blank control. Establishment of celiac disease oxidative stress model was detailed in **Supplementary Methods**. The model cells were stimulated by 5 μmol/L resveratrol for 3 h.

### Mouse Model

The second generation of female C57BL/6N mice (6 weeks old) were used as experiment animals and co-stimulated by gliadin, α-amylase/trypsin inhibitors (ATI) and Polyinosinic:polycytidylic acid (Poly : IC) (**Supplemental Materials**). The model mice were gavaged with 100 mg/kg resveratrol as described by Singh (19).

### Determination of Oxidative Stress Indexes

Mice jejunum tissue were lysed to determine the protein concentration, with subsequent determination of NO content, GSH content, MDA content and SOD enzyme activity using the kit. Others were shown in **Supplemental Methods**.

### Quantitative PCR

RNA was extracted from Caco-2 cells with TRIzol and its concentration was determined by Nanodrop (Thermo Fisher, Massachusetts, USA). The reverse transcribed cNDA samples were subjected to quantitative polymerase chain reaction by StepOnePlus Real-Time PCR instrument (Bio-Rad, California, USA). The relative mRNA expression of the tested gene was calculated using the 2^-△△Ct^ method.

### Trans Epithelial Electrical Resistance of Cells

Before measuring the trans epithelial electrical resistance (TEER), the electrodes of the resistivity meter (Millipore, Massachusetts, USA) were disinfected by soaking in 70% alcohol for 30 min. The electrodes were washed with PBS buffer and left to dry at room temperature. The resistance values were measured by inserting the ends of the resistivity meter into the upper and lower chambers of each well of the Transwell culture plate, with the short pole on the inside and the long pole on the outside. Each well was measured three times by taking points in different directions. The results were recorded (Rt) and the resistance value (R0) of the blank well was also determined. TEER=(Rt-R0)*S (S means the effective membrane area, 1.12cm^2^).

### Monolayer Permeability of Cells

The Caco-2 cells were cultured in the upper chamber of Transwell plates for 2 h using sodium fluorescein, and the cell solution in the lower chamber was collected for absorbance value detection at 490nm. The apparent permeability coefficient was calculated from the standard curve.

### Western Blot Analysis

The samples of Caco-2 cell were lysed on ice. 30 µg protein samples were loaded onto a 10% SDS-PAGE and electrophoresed at constant voltage of 100 V for 1.5 h. The proteins were transferred onto polyvinylidene fluoride membranes and blocked with 5% skimmed milk for 1 h at room temperature and washed with Tris Buffered Saline with 0.1% Tween (TBST), followed by incubation with the TG2 (clone NO. CUB 7402), Occludin (clone NO. OC-3F10) and β-actin (clone NO. 15G5A11/E2) monoclonal antibodies (1:200, 1:1,000 and 1:5,000, respectively, Mouse/IgG1, Thermo Fisher scientific, Massachusetts, USA) at 4°C overnight. After washing with TBST, the membranes were incubated with horseradish-peroxidase conjugated secondary antibodies (1:3,000, Sigma, Missouri, USA) for 1 h at room temperature. Finally, the membranes were visualized using ChemiDocTM Touch (Bio-Rad Laboratories, Inc. California, USA) and quantified by using ImageJ software (NIH, Bethesda, MD).

### RNA-Sequencing

The total RNA from mice jejunal tissue was extracted by Trizol. The RNA was purified, fragmented, reverse transcribed and amplified. The library fragments were enriched using PCR and performed quality control, and then sequenced on Illumina platform. The mouse reference gene (Mus musculus.GRCm38.dna.primary assembly.fa) was searched using Genome in NCBI (its Path was ftp://ftp.ensembl.org/pub/release-101/fasta/mus_musculus/dna/), and the genome database was referenced to Ensembl. Filtered Reads were compared to the C57BL/6 mouse reference genome using HISAT2 software. Differential statistical analysis of gene expression was performed by DESeq. Volcano maps of differentially expressed genes were drawn using the R language ggplots2 package to show gene distribution, gene expression ploidy differences and significance results. Two-way clustering analysis of the concatenated sets and samples of differential genes was performed using the R language Pheatmap package, and the Euclidean method was used to calculate the distance and the Complete Linkage was used for clustering. GO enrichment analyses were performed with the database established by the Gene Ontology Consortium (http://geneontology.org/). KEGG enrichment analyses were performed with the database of Kyoto Encyclopedia of Genes and Genomes (http://www.kegg.jp/). Sequencing service were provided by Personal Biotechnology Co., Ltd. Shanghai, China. The data were analyzed by using the free online platform Personalbio GenesCloud (https://www.genescloud.cn).

### Statistical Analysis

Experimental results were expressed as the mean ± standard deviation (M ± SD) of data. Other values were analyzed by independent samples t-test in IBM SPSS Statistics 24 software with two-tailed analysis, and probability values of p < 0.05 were considered significantly different. Additional methods are shown in the **Supplementary Material**.

## Results

### Protective Effect of Resveratrol Against Oxidative Damage in Caco-2 Cells

Firstly, we established a model of celiac oxidative stress induced by the gluten protein-derived peptide p31-43 in Caco-2 cells. The action of 125 μg/mL p31-43 for 3 h induced up to 30% cell loss of viability compared to the control group (**Supplementary Figure 1A**). Peptide p31-43 at 25 μg/mL and 125 μg/mL concentrations stimulated cells for 24 h increased the intracellular ROS content (**Supplementary Figure 1B**). Then, cells were stimulated with p31-43 peptide fragments for 24 h to detect changes in intracellular SOD, MDA and GSH (**Supplementary Figures 1C-E**). According to the results of SOD, GSH and MDA, the best oxidative stress model for celiac disease was obtained by stimulation with 125 μg/mL p31-43 peptide for 24 h. Meanwhile, stimulation for 3 h at this concentration of peptide was the best working parameter to detect changes in cell viability of this model.

Subsequently, the concentration and treatment time of resveratrol were investigated. Concentrations of resveratrol below 15 μM didn’t cause a decrease in cell viability of Caco-2 cells treated with resveratrol for 24 h (**Supplementary Figure 2A**). Moreover, Caco-2 cells were treated with resveratrol below 15 μM for 3 h, followed by being stimulated with p31-43 peptide at a concentration of 125 μg/mL for 3 h. 5 μmol/L of resveratrol provided optimal recovery of cell viability (**Supplementary Figure 2B**). Then, treatment of cells incubated with 5 μmol/L resveratrol for 3 h reduced intracellular ROS levels, and reduction reached 46% when compared with control (**Supplementary Figure 2C**). Incubation of cells with 5 μmol/L resveratrol for 3 h was the optimal action condition.

**Figure 1:**
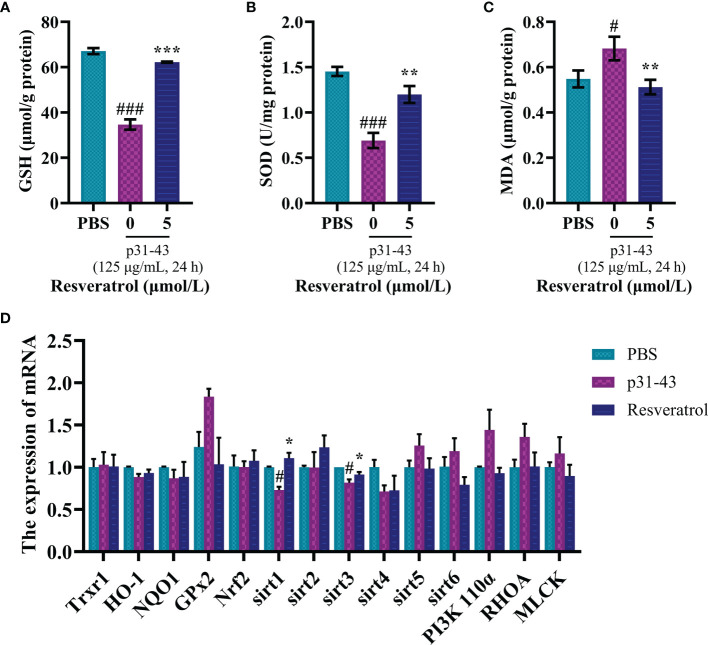
Resveratrol mitigated damage from oxidative stress in cell model for celiac disease. **(A)** GSH content. **(B)** Enzymatic activity of SOD. **(C)** MDA content. **(D)** Expression of antioxidant gene. ^#^Indicated statistically significant differences when compared to the PBS group. ^#^P < 0.05; ^###^P < 0.001. * Indicated statistically significant differences when compared to the p31-43 group. *P < 0.05; **P < 0.01; ***P < 0.001.

The effects of resveratrol on the disruption of redox system in Caco-2 cells were further verified. We found that the stimulated cells with 5 μmol/L resveratrol for 3h could restore the levels of GSH, SOD and MDA in cells (**Figures 1A–C**). Moreover, three signaling pathways related to antioxidant function, Nrf2-ARE (20), PI3K-AKT-mTOR (21) and SIRT (22), were used to investigate critical genes of resveratrol intervention (**Figure 1D**). Treatment of the p31-43 peptide fragment in the blank group caused a decrease in the expression of sirt1 and sirt3 genes in Caco-2 cells, while the expression of these two antioxidant genes was enhanced by resveratrol. Together, all these results suggested that resveratrol could effectively alleviate oxidative damage caused by toxic gliadin peptides in Caco-2 cells, which may be associated with the sirt1/sirt3-related SIRT signaling pathway.

### Alleviative Effect of Resveratrol on the Celiac Toxicity in Caco-2 Cells

**Figure 2:**
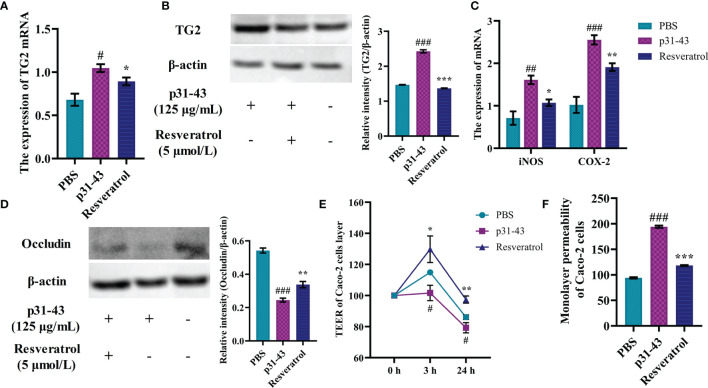
Resveratrol regulated celiac disease-related indicators in Caco-2 cells. **(A)** The expression of TG2 gene. **(B)** The expression of TG2 protein. **(C)** The expression of iNOS/COX-2 gene. **(D)** The content of Occludin protein. **(E)** Monolayer resistance values of Caco-2 cells. **(F)** Monolayer permeability of Caco-2 cells. ^#^Indicated statistically significant differences when compared to the PBS group. ^#^P < 0.05; ^##^P < 0.01; ^###^P < 0.001. * Indicated statistically significant differences when compared to the p31-43 group. *P < 0.05; **P < 0.01; ***P < 0.001.

Caco-2 cells as a model exposed to gluten *in vitro* could simulate many of the celiac toxic features, such as activation and overexpression of intracellular TG2, inter-enterocyte’s tight junction dysfunction and expression of pro-inflammatory cytokines (23). The p31-43 peptide increased intracellular expression of TG2 gene by 80% and elevated intracellular content of TG2 protein relative to the blank group, while intracellular TG2 gene and protein expression were reduced under resveratrol intervention (**Figures 2A, B**). Similarly, intracellular expression of iNOS and COX-2 genes was surged by stimulation of p31-43, and resveratrol reduced expression of iNOS to 69% of p31-43 group and COX-2 to 75% of p31-43 group (**Figure 2C**). The results of expression of tight junction proteins in Caco-2 cells showed that the p31-43 disrupted the tight junctions between Caco-2 monolayers, leading to a decrease in expression of occludin, while resveratrol could effectively inhibit the hypo-expression of occludin (**Figure 2D**). Moreover, the TEER values of Caco-2 cells were elevated and the monolayer permeability coefficients of Caco-2 cell were reduced after the cells were treated with resveratrol when compared with p31-43 group (**Figures 2E, F**). Collectively, these findings indicated that resveratrol could effectively mitigate the celiac toxicity actions on Caco-2 cells caused by the p31-43 peptide.

### Intervention Effect of Resveratrol on Clinical Symptoms in the Mice With Celiac Disease

At first, according to the mouse model of Zevallos et al. (18), we established a mouse model for celiac disease. The results and details of this model were shown in **Supplementary Figure 3A**. Celiac disease, as an autoimmune-mediated intestinal inflammation, mostly presented with symptoms associated with intestinal malabsorption, which included (severe) chronic diarrhea and weight loss (24). In our research, the body weight of ATI+Poly : IC+Gliadin group and ATI+DSS+Gliadin group were gradually decreased from the 3 day when compared to control group (**Supplementary Figure 3B**). Water consumption in mice with DSS in their drinking water was higher in the first three days than that in the control group, and the ATI+Gliadin and ATI+CT groups showed an increase in water consumption after the 7th day (**Supplementary Figure 3C**). Moreover, patients with celiac disease often developed symptoms of extraintestinal related diseases, such as the common herpes-like dermatitis, because of the spread of adaptive immune responses outside mucosal tissues of intestine (25). So, the experimental mice were scored for symptoms during the 4 h excitation phase (**Supplementary Figures 3D, E**). 5 mice in the ATI+Gliadin group exhibited diarrhea or reduced activity. Symptoms of tremor or cramp in mice in the ATI+Poly : IC group increased from 2 to 4 mice after the addition of gliadin to their diet, and the number of scratches appeared to decrease. In the ATI+DSS group, 4 mice exhibited tremor and 1 exhibited tremor/blood in stool, whereas after the addition of gliadin, all mice exhibited tremor/blood in stool, along with increased scratching.

Celiac disease was an inflammatory disease of the intestine which occurred mainly in the small intestine, and sections of ileal and jejunal tissue were selected for HE sections. The ATI+Poly : IC group with the addition of Poly : IC showed more atrophy and damage to the villi than that in the ATI group, and the ATI+Poly : IC+Gliadin group with the addition of gliadin showed further atrophy and damage to the intestinal villi as well as inflammatory cell infiltration (**Supplementary Figure 4A**). The ATI+Poly : IC+Gliadin group showed the most severe structural damage of the intestinal villi in both ileum and jejunum of mice.

In the small intestinal tissues of celiac disease patients, a subpopulation of Th1 cells rises dramatically and predominates in the onset of the disease, which eventually progresses to intestinal inflammation mediated by Th1 immune cells (26). As shown by the ratio of Th1/Th2 type immune cells in the mesenteric lymph nodes of mouse, the Th1/Th2 ratio in the MLN of mice in the Gliadin group, ATI+Gliadin group and ATI+Poly : IC+Gliadin group was higher only after stimulation with Gliadin in the diet (**Supplementary Figure 4B**). Moreover, the Th1/Th2 ratio was decreased in the ATI+DSS+Gliadin group after addition of gliadin. The expression levels of relevant inflammatory factors secreted by Th1 and Th2 type immune cells in the jejunum of mice were further determined. The ATI+Poly : IC+Gliadin group had the highest expression levels of IL-1β, IL-2 and TNF-α among all groups containing gliadin, while IL-1β and TNF-α were increased in this group when compared to normal mouse (**Supplementary Figure 4C**). The expression of IL-2 and TNF-α in the ATI+CT+Gliadin group were elevated after addition of gliadin but was far from that in the ATI+Poly : IC+Gliadin group. However, Th2 related inflammatory factors mostly showed inhibition of expression after adding gliadin. The expression of IL-4 and IL-6 were reduced in the ATI+Poly : IC+Gliadin group after stimulation with gliadin, while the ATI+CT+Gliadin group showed the opposite trend (**Supplementary Figure 4D**). The expression level of IL-10, which showed a similar trend to that of Th2, was lower in the ATI+Poly : IC+Gliadin group after addition of gliadin. Among them, the expression of IL-10 was decreased in the ATI+Poly : IC+Gliadin group when compared with the Gliadin group and ATI+Gliadin group.

These results indicated that the use of CT and DSS didn’t produce symptoms associated with celiac disease in mice under the gliadin stimulation. The TG2 played unique features in the pathogenesis of celiac disease by modifying gluten peptides, which in turn exacerbated the inflammation (27). The AOD values of TG2 in jejunal tissues of mice in both ATI+Gliadin and ATI+Poly : IC+Gliadin groups were found to be higher than those in the Gliadin group, and the highest values were found in the ATI+Poly : IC+Gliadin group (**Supplementary Figure 4E**). In summary, the symptoms and indices of mice in the ATI+Poly : IC+Gliadin group (APG group) were the closest to those of celiac disease, and it could be considered as an animal model of celiac disease for subsequent experiments.

**Figure 3:**
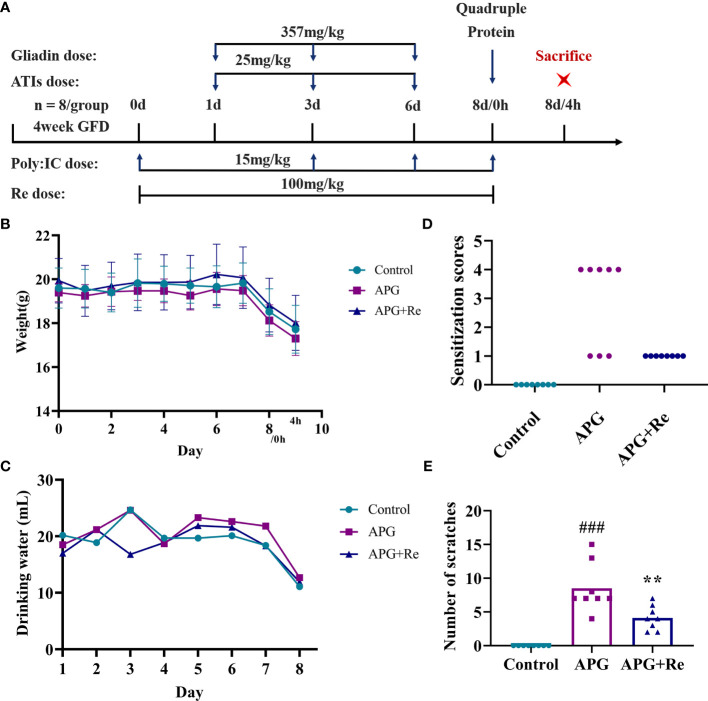
Resveratrol intervenes in the clinical symptoms of the mice with celiac disease. **(A)** Nutritional intervention of resveratrol in the mice with celiac disease. **(B)** Weight of mice. **(C)** Water consumption of mice. **(D)** Clinical symptoms of mice. **(E)** Number of scratches in mice. ^#^Indicated statistically significant differences when compared to the control group. ^###^P < 0.001. * Indicated statistically significant differences when compared to the APG group. **P < 0.01. Symptom scoring: (0) No symptoms; (1) Scratching nose and mouth; (2) Swelling around the eyes and mouth, diarrhea, reduced activity or walking in place, higher breathing rate; (3) Shortness of breath, wheezing, blue rash around the mouth and tail; (4) Loss of consciousness, tremors or cramps, blood in stool; (5) Death by shock.

Then, we conducted the resveratrol intervention study in this mice model (**Figure 3A**). The body weights of the APG+Re group were higher than those of the APG group starting from 5 day under resveratrol (**Figure 3B**). The water consumption of mice in the APG+Re group decreased only at 3 day, and there were no significant differences between the groups at other days (**Figure 3C**). Five of the eight mice in the APG group exhibited tremors relative to control group, and scratches were higher. However, after the dietary intervention with resveratrol, the mice in APG+Re group exhibited scratching only and scratches were lower (**Figures 3D, E**). Together, all these results evidenced that resveratrol could alleviate the clinical symptoms of celiac disease in mice with intestinal inflammation.

### Protective Effect of Resveratrol on Oxidative Damage in the Intestine of the Mice With Celiac Disease

**Figure 4:**
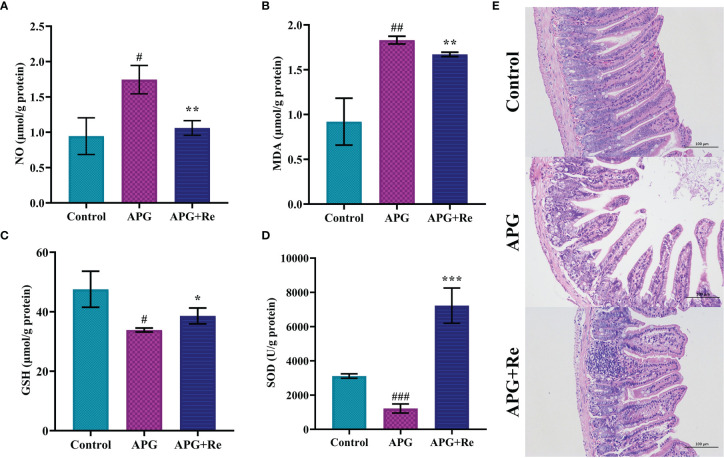
Resveratrol mitigated damage from oxidative stress in the mice with celiac disease. **(A)** NO content in jejunal tissue. **(B)** MDA content in jejunal tissue. **(C)** GSH content in jejunal tissue. **(D)** Enzymatic activity of SOD in jejunal tissue. **(E)** Intestine histological sections of jejunum stained with hematoxylin and eosin stain from mice. ^#^Indicated statistically significant differences when compared to the control group. ^#^P < 0.05; ^##^P < 0.01; ^###^P < 0.001. * Indicated statistically significant differences when compared to the APG group. *P < 0.05; **P < 0.01; ***P < 0.001. **(E)** are the fields of view at 200x lens.

We found that NO and MDA content in supernatant of jejunal tissue lysates were increased in mice after treatment with ATI+Poly : IC+Gliadin, while intervention with resveratrol could reduce it (**Figures 4A, B**). In contrast, GSH content and activity of SOD in supernatant of jejunal tissue lysate were reduced after ATI+Poly : IC+Gliadin action in mice, while it was increased by intervention of resveratrol (**Figures 4C, D**). Moreover, the jejunum of mice didn’t show severe villi damage, while crypt hyperplasia and inflammatory cell infiltration were similar to the APG group after resveratrol dietary intervention (**Figure 4E**). Collectively, resveratrol could effectively alleviate oxidative stress and protect damage of the intestine in the mice with celiac disease.

### Alleviative Effect of Resveratrol on Toxic Signs in the Mice With Celiac Disease

**Figure 5:**
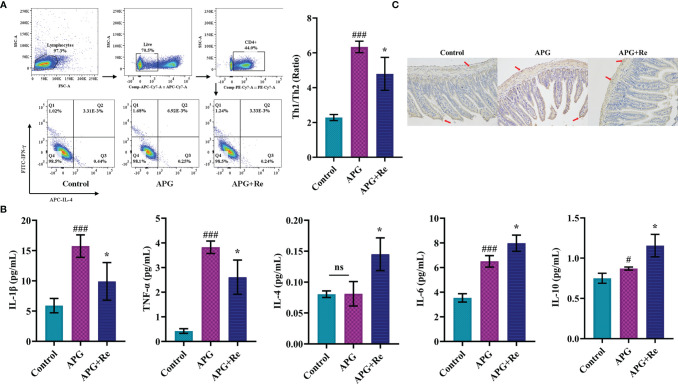
Resveratrol modified T cell function in mesenteric lymph nodes and jejunum of celiac mice and inhibited expression of characteristic autoantigens in the jejunum. **(A)** The ratios of Th1/Th2 cells in mesenteric lymph nodes of mice. **(B)** The expression level of Th1 related inflammatory factors (IL-1β and TNF-α), Th2 related inflammatory factors (IL-4 and IL-6) and IL-10 in the lysate of jejunum tissue. **(C)** Immunohistochemistry analysis of tissue transglutaminase in jejunum tissue sections. ^#^Indicated statistically significant differences when compared to the control group. ^#^P < 0.05; ^###^P < 0.001. * Indicated statistically significant differences when compared to the APG group. *P < 0.05. **(C)** were the fields of view at 200x lens. ns, no significant differences.

We further determined by subpopulation of Th cells in MLN, and the Th1/Th2 ratio in MLN of mouse in APG group was significantly increased after co-stimulation with ATI+Poly : IC+Gliadin, while the Th1/Th2 ratio in MLN of APG+Re group was decreased after intervention with resveratrol when compared to APG group (**Figure 5A**). From the perspective of nutritional intervention, resveratrol should act through inhibiting the overexpression of Th1 cell function and slowing down the depression of Th2 cell function, which would promote a state of dynamic balance between Th1 and Th2 subpopulations. The expression of IL-1β and TNF-α were both elevated in the intestine APG group when compared to the control group, which was consistent with the previous results. In contrast, the expression of IL-1β and TNF-α were both lower in the APG+Re group compared to the APG group after intervention with resveratrol (IL-1β and TNF-α, **Figure 5B**). The APG and control groups didn’t show differences that were consistent with the previous result of IL-4. Moreover, the APG+Re group showed higher expression of IL-4 after addition of resveratrol (IL-4, **Figure 5B**). Similarly, dietary intervention with resveratrol could increase the expression of IL-6 and IL-10 in the APG group (**Figure 5B**). Additionally, the content of TG2 in the jejunal tissue of mice of APG group was higher than that of control group, which was consistent with the previous results. The values were lower after intervention with resveratrol when compared with the APG group (**Figure 5C** and **Supplementary Figure 5**). Together, above results indicated that resveratrol could effectively reduce the celiac toxicity in the model mice.

### Regulatory Role of Resveratrol on Characteristic Genes and Pathways in the Mice With Celiac Disease

**Figure 6:**
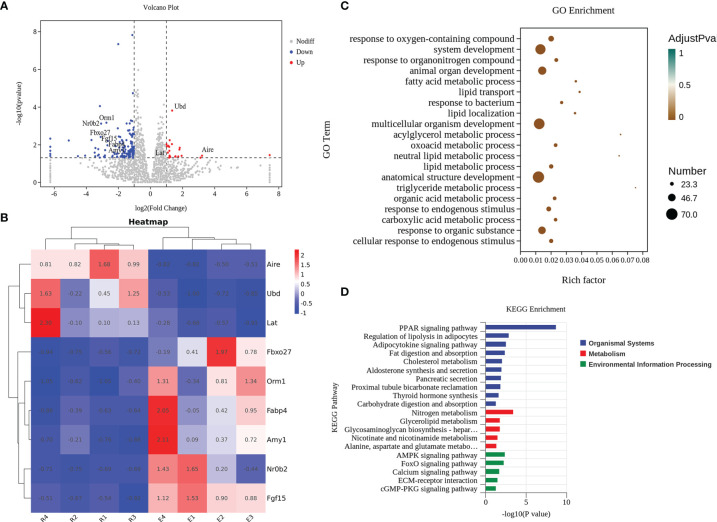
Resveratrol effectively acted by altering characteristic genes and pathways in the jejunal tissue of mice. **(A)** Volcano plot of differential genes. **(B)** Cluster analysis of characteristic genes. E: The celiac disease mouse model (APG group). R: The intervention of resveratrol (APG+Re group). **(C)** GO enrichment analyses. **(D)** KEGG enrichment analyses.

Finally, we aimed to determine why a dietary intervention with resveratrol alleviates the occurrence of celiac disease. We analyzed changes in signature genes and related pathways after resveratrol intervention. After quality control and expression analysis, we mapped volcanoes for differential genes and selected six down-regulated genes (Orm1, Nr0b2, Fbxo27, Fgf15, Fabp4 and Amy1) and three up-regulated genes (Ubd, Lat, and Aire) (**Figure 6A**). Further plotting the variability of these nine genes as a heatmap, the Fgf15 and Nr0b2, Aire and Ubd showed the most significant performance in the up-regulated and down-regulated genes, respectively (**Figure 6B**). Meanwhile, the differentially expressed genes after intervention with resveratrol were classified. By GO enrichment analysis, the differential genes were mainly involved in biological processes such as reactions with oxygenated compounds, phylogeny and reactions with organic nitrogen compounds (**Figure 6C**). KEGG enrichment analysis indicated that the differential genes were mainly related to PPAR signaling pathway, nitrogen metabolism, AMPK signaling pathway and FoxO signaling pathway (**Figure 6D**).

## Discussion

Impairment of antioxidant defenses would lead to oxidative stress and damage in the intestinal and extraintestinal tissues of patients with celiac disease (28). In our cell model experiments, it was found that the ingestion of gluten peptide p31-43 or gluten not only lead to an increased oxidative stress response, but also aggravate intestinal inflammation. However, resveratrol, as a natural antioxidant, had efficient antioxidant activity and was able to modulate oxidative stress damage in the intestine (29). Meanwhile, Mayangsari et al. (30) discovered that resveratrol reduced the inflammatory factor formation in the intestine and improved intestinal barrier damage in a mouse model of colitis and an intestinal epithelial cell model. We speculated that resveratrol may inhibit oxidative stress to ameliorate inflammatory damage of intestine and thus alleviate the symptoms associated with celiac disease. The work in the Caco-2 cell model supported that nutritional intervention with resveratrol significantly improved cell viability and mitigates oxidative damage. Resveratrol abrogated the adverse effects of the gluten protein-derived peptide p31-43 and inhibited the overexpression of TG2 and inflammatory mediators iNOS and COX-2, thereby ameliorating dysfunction of cell barriers. This was consistent with Alharris’ study (31) that resveratrol could directly enhance the solidity of the epithelial barrier against the LPS-induced barrier disruption by increasing the expression of tight junction proteins. We identified an association of oxidative stress damage with the pathogenesis of celiac disease, where barrier function of the intestinal epithelium was disrupted by the upregulated oxidative stress in the tissues, which in turn promoted the development of the disease. Accordingly, this work indicated that resveratrol could play an efficient protective role in the cell model for celiac disease.

Resveratrol could provide multitargeted treatment for multiple chronic diseases (32) and autoimmune diseases (33), including the modulation of multiple cell signaling molecules, such as cytokines, NF-kB, intercellular adhesion molecule, protein kinase, sirtuin-type-1, cyclooxygenase-2, and so on. In the C57BL/6 mouse model of celiac disease, our study supported that nutritional intervention with resveratrol significantly improved clinical symptoms and reduced oxidative damage in mice, while regulating Th cell subpopulation toward Th2 differentiation which in turn inhibited Th1 immune cell function overexpression and relieved Th2 immune cell function under-expression, as well as decreasing TG2 content in jejunal tissue. Here, resveratrol plays a critical role in alleviating celiac disease not only by inhibiting the occurrence of oxidative stress to improve intestinal damage in celiac mice, but also by effectively modulating the immune response of organism.

Resveratrol was able to confer apoptotic resistance to oxidative stress through regulation of SIRT (34) and improve mitochondrial function to prevent metabolic diseases (35). Our work in cells model demonstrated that resveratrol could inhibit the overexpression of iNOS and COX-2 by upregulating the sirt1 and sirt3 genes. The iNOS and COX-2 genes could directly regulate the balance of intracellular nitrogen and oxygen to maintain the progression of oxidative stress and inflammatory responses, which were usually influenced by NF-kB and PPAR-γ signaling pathways. The NF-κB signaling pathway has an important role in inflammatory bowel disease, which induces the secretion of inflammatory cytokines (TNF-α, IL-1β, IL-12) and upregulates the expression levels of pro-inflammatory factors COX2 and oxidative stress markers (36). Numerous cellular models and animal experiments have shown that resveratrol can reduce expression levels of COX2 and significantly inhibit NF-κB signaling pathway activity (37). Singh et al. (19) demonstrated that resveratrol could abrogate DSS-induced colitis by down-regulating the activation of NF-κB, reducing the levels of associated inflammatory factors, and inhibiting the expression of COX-2.

Moreover, nutritional intervention of resveratrol could affect the differential expression of Aire, Ubd, Fgf15 and Nr0b2 genes in the intestine of the mice with celiac disease. Here, we elaborated a resveratrol-mediated association between differentially expressed genes and their specific roles. Aire (autoimmune regulator) is a modulator of the body’s autoimmunity and its disruption of function (such as Aire deficiency or disabled) can lead to autoimmune diseases in humans and mice (38). Most studies on Aire focused on thymus and peripheral lymphocytes, while our study indicated significant upregulation of the Aire gene in mouse jejunum after the intervention of resveratrol. Aire may induce mixed expression of tissue-specific antigens (TSA) to induce autoimmune tolerance for celiac disease (39). In addition, Aire could physically bind to sirt1, which deacetylated its lysine residues, thereby activating Aire transcription (40). In contrast, Ubd (Ubiquitin-binding domain) was used as a ubiquitin-like protein modifier to target proteins for proteasomal degradation, regulation of immune signaling pathways and DNA repair (41). The significant rise of Ubd gene in mouse intestine after resveratrol intervention will specifically inhibit PPAR-related proteins from proteasomal degradation and thus functionally regulate PPAR signaling. Moreover, Fgf15 (Fibroblast growth factor 15) was a gut-derived hormone that acted on the liver to inhibit the synthesis of bile acids and to facilitate the distribution of nutrients after meals (42). Bile acids (BAs) were essential in absorption of dietary lipids and metabolism of cholesterol catabolism (43). Huang et al. (44) found that theabrownin could reduce hepatic cholesterol and decrease adipogenesis by inhibiting FXR-FGF15 signaling in the intestine. Chen et al. (45) found that resveratrol modulated atherosclerosis (AS) through remodeling of the gut microbiota and downregulating the enterohepatic FXR-FGF15 axis. The nuclear receptor subfamily 0 group B member 2 (NR0B2, also called SHP) was normally highly expressed in the liver and intestine, having intestinal functions that inhibit cholesterol absorption and acting as a regulator of bile acids (46). Fgf15/19 and Fgf15/19-activated Nr0b2 play a suppressive role in the transcription of adipogenesis that could act as nutrient signals to regulate nutrient uptake (47). The intervention of resveratrol in this study significantly reduced the expression of Fgf15 and Nr0b2 genes in the intestine of mice, which may restore intestinal absorption of nutrients by regulating the secretion of bile acids. Together, it may be postulated that resveratrol could alleviate the overreaction caused by autoimmunity through up-regulation of Aire and Ubd genes and promote intestinal absorption of nutrients through down-regulation of Fgf15 and Nr0b2 genes, ultimately alleviating the intestinal damage caused by wheat gluten.

**Figure 7:**
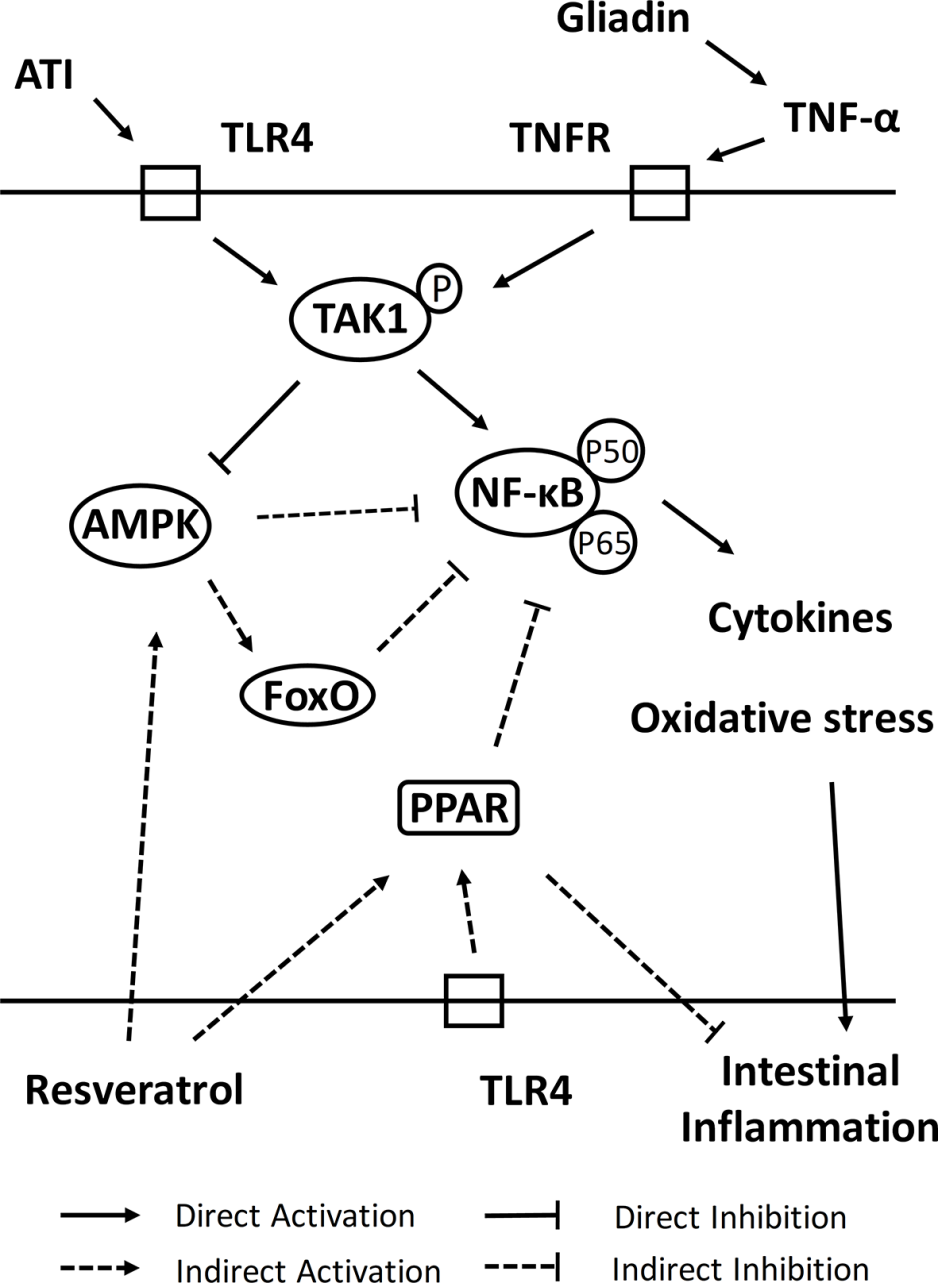
The mechanism of resveratrol intervention on intestinal damage of celiac disease.

Furthermore, we have explored associations of resveratrol-activated related pathways and its role in recovery from intestinal damage (**Figure 7**). PPAR is the peroxisome proliferator-activated receptors that promoted the regulation of energy production, lipid metabolism, cell differentiation and ligand-dependent transcription of inflammation-targeted genes (48). In intestinal epithelial cells, accumulation of the wheat gluten peptide p31-43 lead to elevate level of reactive oxygen species (ROS) and downregulate level of tissue transglutaminase-mediated PPAR-γ (11). PPAR-γ was expressed not only in intestinal epithelial cells (IEL) but also in intestinal macrophages and T cells, where it inhibited NF-κB signaling and COX-2 production and thus restored mucosal damage (49). Among them, NF-κB (nuclear factor-κB) was a commonly expressed transcription factor involved in the regulation of innate immunology, inflammation and metabolic stress, being a key transcription factor for the pro-inflammatory factor TNF-α (50). In addition, PPAR-α and PPAR-β inhibited macrophage activation and suppress NF-κB signaling, thereby reducing the production of inflammatory factors (TNF-α, IL-1β and IFN-γ) (51). PPAR-α could promote expression of IL-10 and inhibit expression of pro-inflammatory factors, which in turn inhibited Th1/Th17 differentiation (52), which was consistent with intervention results of resveratrol in this study. Similarly, PPAR-β altered differentiation of the T cell population, resulting in suppression of Th1/Th17 cell subpopulation (decreases expression of inflammatory factors such as IFN-γ, IL-17, and IL-12) and polarization toward Th2 cell subpopulation (elevates expression of IL-4 and IL-10) (53). Activation of PPAR-β also elevated SOD and catalase activities and reduced thioredoxin levels, which consequently antagonizes multiple pro-inflammatory pathways (54). Moreover, binding of ligand effectively prevented degradation of ubiquitination-induced PPAR-β, and Ubd gene which was significantly upregulated by resveratrol will help to regulate protein levels of PPAR-β (55). AMPK acted as a sensor of energy conservation and often played a key role in antioxidant defense of cells and in regulating cellular activities such as cell proliferation, cycle progression and apoptosis (56). Activation of AMPK in macrophages decreased accumulation of ROS, NO and COX-2, while inhibiting NF-κB activity, which in turn regulated inflammatory responses (such as upregulation of IL-10 levels and downregulation of TNF-α and IL-1β levels) and process of T cell activation (such as development of Th1/Th17 cells) (57, 58). In their study of resveratrol intervention in Acute Respiratory Distress Syndrome (ARDS), Alghetaa et al. (59) found that resveratrol could lead to a decrease in effector T cells by increasing apoptosis of T cells. In the pseudo-starvation view, AMPK activators could alter program of metabolic involvement in inflammatory signaling, allowing AMPK to efficiently regulate catabolic pathways of energy and thus limit inflammatory responses (60). We speculated that activation of AMPK by resveratrol in intestine of mice was compliant with pseudo-starvation-restricted inflammatory metabolism. In addition, pattern recognition receptors (such as Toll-like receptor) that were stimulated in intestinal epithelial cells could activate IL-1β and lead to inactivation of AMPK to induce death of inflammatory cells and consequently impair barrier function of intestine (61). ATI exerted these effects in the intestine *via* TLR4-MD2-CD14, which further exacerbated the toxic effects caused by gluten (62). The intervention of resveratrol then made activation of TLR4 to re-encode macrophages for an anti-inflammatory phenotype, while exerting an anti-inflammatory effect of diet. In addition, cancer cells will downregulate AMPK by ubiquitinating degradation of AMPKα1 (63), and thus we speculated that Ubd gene also had a protective effect on AMPK signaling. Forkhead box transcription factor O (FoxO) was involved in a variety of biological processes *in vivo* that could modulate intestinal mucosal immunity by inhibiting expression of inflammatory cytokines (such as down-regulation of TNF-α and IFN-γ) and restore intestinal function by inhibiting NF-κB (64). Furthermore, AMPK directly regulated activation of FoxO, which decreased expression of ROS and increased expression of SOD, catalase and sestrin in order to increase resistance to oxidative stress (65). Taken together, these results provided strong evidence that resveratrol could regulate oxidative stress, inflammation and immune responses in intestine by activating PPAR, AMPK and FoxO signaling pathways, thereby alleviating intestinal damage in celiac disease mice.

In summary, we demonstrated the association between oxidative stress and inflammatory response in the pathogenesis of celiac disease from current *in vivo* and *in vitro* models. Moreover, using resveratrol as an entry point for intervention, we demonstrated that resveratrol could regulate development of autoimmunity through up-regulation of Aire and Ubd genes and promote nutrient absorption in intestine through down-regulation of Fgf15 and Nr0b2 genes, as well as play a role in regulating complex response system of oxidative stress, inflammatory response and immune response in intestine by activating PPAR, AMPK and FoxO signaling pathways, thus effectively alleviating the intestinal symptoms of celiac disease. It should be interesting to determine whether other natural antioxidants or activators have similar effects and mechanisms to resveratrol. In conclusion, our data supported that dietary intake of resveratrol could serve as a novel approach to prevent the development of celiac disease.

## Data Availability Statement 

The data presented in the study are deposited in the NCBI (National Center for Biotechnology Information, https://www.ncbi.nlm.nih.gov/) repository, accession number PRJNA809515.

## Ethics Statement

The animal study was reviewed and approved by The Experimental Animal Ethics Committee of Jiangxi University of Traditional Chinese Medicine.

## Author Contributions

TY and YX conceived the study, designed and performed experiments, and wrote the manuscript. JY, JG, and ZX analyzed the data. YW and HC wrote and revised the main manuscript. All authors contributed to the article and approved the submitted version.

## Funding

This work was supported by the International Science & Technology Cooperation Program of China (No. 2013DFG31380), the Jiangxi Province funding program (No. 20212BAB205034), and Central Government Guide Local Special Fund Project for Scientific and Technological Development of Jiangxi Province (No. 20212ZDD02008).

## Conflict of Interest

The authors declare that the research was conducted in the absence of any commercial or financial relationships that could be construed as a potential conflict of interest.

## Publisher’s Note

All claims expressed in this article are solely those of the authors and do not necessarily represent those of their affiliated organizations, or those of the publisher, the editors and the reviewers. Any product that may be evaluated in this article, or claim that may be made by its manufacturer, is not guaranteed or endorsed by the publisher.

## Supplementary Material

Supplementary Figure 1 Gluten protein-derived peptide p31-43 induced Caco-2 cells to construct a celiac oxidative stress model. **(A)** Changes of caco-2 cells activity. **(B)** The accumulation of intracellular ROS. Changes in SOD enzyme activity **(C)**, GSH content **(D)** and MDA content **(E)** in Caco-2 cells after 24 h of P31-43 peptide stimulation. * indicated statistically significant differences when compared to the PBS group. *P < 0.05; **P < 0.01; ***P < 0.001.

Supplementary Figure 2 Determination of resveratrol concentration and pretreatment time. **(A)** Safety concentrations of resveratrol in Caco-2 cells. * indicated statistically significant differences when compared to the PBS group. *P < 0.05; ***P < 0.001. **(B)** Effects of resveratrol on cell viability loss induced by p31-43. **(C)** Regulation of resveratrol on intracellular ROS accumulation in Caco-2 cells. # indicated statistically significant differences compared to the PBS group. ##P < 0.01; ###P < 0.001. * indicated statistically significant differences when compared to the control group. *P < 0.05; **P < 0.01; ***P < 0.001.

Supplementary Figure 3 Construction of a mouse model for celiac disease. **(A)** Experimental protocol for model construction. **(B)** Weight of mice. **(C)** Water consumption of mice. **(D)** Clinical symptoms of mice. **(E)** Number of scratches in mice. *P < 0.05; **P < 0.01; ***P < 0.001. Symptom scoring: (0) No symptoms; (1) Scratching nose and mouth; (2) Swelling around the eyes and mouth, diarrhea, reduced activity or walking in place, higher breathing rate; (3) Shortness of breath, wheezing, blue rash around the mouth and tail; (4) Loss of consciousness, tremors or cramps, blood in stool; (5) Death by shock.

Supplementary Figure 4 Characteristic indicators of the mouse model for celiac disease. **(A)** Intestine histological sections of ileum (above) and jejunum (below) stained with hematoxylin and eosin stain from mice. **(B)** The ratios of Th1/Th2 cells in mesenteric lymph nodes of mouse. **(C)** The expression level of Th1 related inflammatory factors (IFN-γ, IL-1β, IL-2, TNF-α), **(D)** Th2 related inflammatory factors (IL-4, IL-6) and IL-10 in the lysate of jejunum tissue. **(E)** Immunohistochemistry analysis of tissue transglutaminase in jejunum tissue sections. Different alphabets of a-e indicated statistically significant differences (p < 0.05). **(A)** were the fields of view at 200x lens. I: Control group; II: Gliadin group; III;: ATI group; IV: ATI+Gliadin group; V: ATI+CT group; VI: ATI+CT+Gliadin group; VII: ATI+Poly : IC group; VIII: ATI+Poly: IC+Gliadin group; IX: ATI+DSS group; X: ATI+DSS+Gliadin group. Figure E were the fields of view at 200x lens. i: Control group; ii: Gliadin group; iii: ATI group; iv: ATI+Gliadin group; v: ATI+Poly : IC group; vi: ATI+Poly : IC+Gliadin group.

Supplementary Figure 5 Average optical density analysis of tissue transglutaminase in jejunum tissue sections.

## Abbreviations

CD, Celiac disease; TG2, the tissue transglutaminase; Gli, gliadin; ATI, α-amylase/trypsin inhibitors; Poly : IC, Polyinosinic:polycytidylic acid; Re, resveratrol.

## References

[1] Pecora F Persico F Gismondi P Fornaroli F Iuliano S de’Angelis GL. Gut Microbiota in Celiac Disease: Is There Any Role for Probiotics? Front Immunol (2020) 11:957. doi: 10.3389/fimmu.2020.00957 32499787 PMC7243837

[2] Caio G Volta U Sapone A Leffler DA De Giorgio R Catassi C. Celiac Disease: A Comprehensive Current Review. BMC Med (2019) 17(1):142. doi: 10.1186/s12916-019-1380-z 31331324 PMC6647104

[3] Mesin L Sollid LM Di Niro R. The Intestinal B-Cell Response in Celiac Disease. Front Immunol (2012) 3:313. doi: 10.3389/fimmu.2012.00313 23060888 PMC3463893

[4] Abadie V Kim SM Lejeune T Palanski BA Ernest JD Tastet O. Il-15, Gluten and Hla-Dq8 Drive Tissue Destruction in Coeliac Disease. Nature (2020) 578(7796):600–4. doi: 10.1038/s41586-020-2003-8 PMC7047598 32051586

[5] Sarna VK Lundin KEA Mørkrid L Qiao S-W Sollid LM Christophersen A. Hla-Dq-Gluten Tetramer Blood Test Accurately Identifies Patients With and Without Celiac Disease in Absence of Gluten Consumption. Gastroenterology (2018) 154(4):886–96.e6.. doi: 10.1053/j.gastro.2017.11.006 29146521

[6] Diaz-Castro J Muriel-Neyra C Martin-Masot R Moreno-Fernandez J Maldonado J Nestares T. Oxidative Stress, DNA Stability and Evoked Inflammatory Signaling in Young Celiac Patients Consuming a Gluten-Free Diet. Eur J Nutr (2020) 59(4):1577–84. doi: 10.1007/s00394-019-02013-5 31144026

[7] Ferretti G Bacchetti T Masciangelo S Saturni L. Celiac Disease, Inflammation and Oxidative Damage: A Nutrigenetic Approach. Nutrients (2012) 4(4):243–57. doi: 10.3390/nu4040243 PMC3347005 22606367

[8] Gómez Castro MF Miculán E Herrera MG Ruera C Perez F Prieto ED. P31-43 Gliadin Peptide Forms Oligomers and Induces Nlrp3 Inflammasome/Caspase 1- Dependent Mucosal Damage in Small Intestine. Front Immunol (2019) 10:31. doi: 10.3389/fimmu.2019.00031 30761127 PMC6363691

[9] Trynka G Wijmenga C van Heel DA. A Genetic Perspective on Coeliac Disease. Trends Mol Med (2010) 16(11):537–50. doi: 10.1016/j.molmed.2010.09.003 20947431

[10] Dinallo V Marafini I Di Fusco D Di Grazia A Laudisi F Dwairi R. Protective Effects of Aryl Hydrocarbon Receptor Signaling in Celiac Disease Mucosa and in Poly I:C-Induced Small Intestinal Atrophy Mouse Model. Front Immunol (2019) 10:91. doi: 10.3389/fimmu.2019.00091 30778350 PMC6369162

[11] Luciani A Villella VR Vasaturo A Giardino I Pettoello-Mantovani M Guido S. Lysosomal Accumulation of Gliadin P31-43 Peptide Induces Oxidative Stress and Tissue Transglutaminase-Mediated Ppargamma Downregulation in Intestinal Epithelial Cells and Coeliac Mucosa. Gut (2010) 59(3):311–9. doi: 10.1136/gut.2009.183608 19951908

[12] Itzlinger A Branchi F Elli L Schumann M. Gluten-Free Diet in Celiac Disease-Forever and for All? Nutrients (2018) 10(11):1796. doi: 10.3390/nu10111796 PMC6267495 30453686

[13] Spaenij-Dekking L Kooy-Winkelaar Y van Veelen P Drijfhout JW Jonker H van Soest L. Natural Variation in Toxicity of Wheat: Potential for Selection of Nontoxic Varieties for Celiac Disease Patients. Gastroenterology (2005) 129(3):797–806. doi: 10.1053/j.gastro.2005.06.017 16143119

[14] Theiss AL Vijay-Kumar M Obertone TS Jones DP Hansen JM Gewirtz AT. Prohibitin Is a Novel Regulator of Antioxidant Response That Attenuates Colonic Inflammation in Mice. Gastroenterology (2009) 137(1):199-208, 208.e1–6. doi: 10.1053/j.gastro.2009.03.033 PMC2788399 19327358

[15] Chimento A De Amicis F Sirianni R Sinicropi MS Puoci F Casaburi I. Progress to Improve Oral Bioavailability and Beneficial Effects of Resveratrol. Int J Mol Sci (2019) 20(6):1381. doi: 10.3390/ijms20061381 PMC6471659 30893846

[16] Luca SV Macovei I Bujor A Miron A Skalicka-Woźniak K Aprotosoaie AC. Bioactivity of Dietary Polyphenols: The Role of Metabolites. Crit Rev Food Sci Nutr (2020) 60(4):626–59. doi: 10.1080/10408398.2018.1546669 30614249

[17] Malaguarnera L. Influence of Resveratrol on the Immune Response. Nutrients (2019) 11(5):946. doi: 10.3390/nu11050946 PMC6566902 31035454

[18] Zevallos VF Raker V Tenzer S Jimenez-Calvente C Ashfaq-Khan M Rüssel N. Nutritional Wheat Amylase-Trypsin Inhibitors Promote Intestinal Inflammation *Via* Activation of Myeloid Cells. Gastroenterology (2017) 152(5):1100–13.e12. doi: 10.1053/j.gastro.2016.12.006 27993525

[19] Singh UP Singh NP Singh B Hofseth LJ Price RL Nagarkatti M. Resveratrol (Trans-3,5,4’-Trihydroxystilbene) Induces Silent Mating Type Information Regulation-1 and Down-Regulates Nuclear Transcription Factor-Kappab Activation to Abrogate Dextran Sulfate Sodium-Induced Colitis. J Pharmacol Exp Ther (2010) 332(3):829–39. doi: 10.1124/jpet.109.160838 PMC2835444 19940103

[20] Kumar H Kim I-S More SV Kim B-W Choi D-K. Natural Product-Derived Pharmacological Modulators of Nrf2/Are Pathway for Chronic Diseases. Nat Prod Rep (2014) 31(1):109–39. doi: 10.1039/c3np70065h 24292194

[21] Kong D Zhang Z Chen L Huang W Zhang F Wang L. Curcumin Blunts Epithelial-Mesenchymal Transition of Hepatocytes to Alleviate Hepatic Fibrosis Through Regulating Oxidative Stress and Autophagy. Redox Biol (2020) 36:101600. doi: 10.1016/j.redox.2020.101600 32526690 PMC7287144

[22] Dai H Sinclair DA Ellis JL Steegborn C. Sirtuin Activators and Inhibitors: Promises, Achievements, and Challenges. Pharmacol Ther (2018) 188:140–54. doi: 10.1016/j.pharmthera.2018.03.004 PMC6342514 29577959

[23] van Bergen J Mulder CJ Mearin ML Koning F. Local Communication Among Mucosal Immune Cells in Patients With Celiac Disease. Gastroenterology (2015) 148(6):1187–94. doi: 10.1053/j.gastro.2015.01.030 25623043

[24] van Wanrooij RLJ Bontkes HJ Neefjes-Borst EA Mulder CJ Bouma G. Immune-Mediated Enteropathies: From Bench to Bedside. J Autoimmun (2021) 118:102609. doi: 10.1016/j.jaut.2021.102609 33607573

[25] Leffler DA Green PHR Fasano A. Extraintestinal Manifestations of Coeliac Disease. Nat Rev Gastroenterol Hepatol (2015) 12(10):561–71. doi: 10.1038/nrgastro.2015.131 26260366

[26] Di Sabatino A Pickard KM Gordon JN Salvati V Mazzarella G Beattie RM. Evidence for the Role of Interferon-Alfa Production by Dendritic Cells in the Th1 Response in Celiac Disease. Gastroenterology (2007) 133(4):1175–87. doi: 10.1053/j.gastro.2007.08.018 17919493

[27] Voisine J Abadie V. Interplay Between Gluten, Hla, Innate and Adaptive Immunity Orchestrates the Development of Coeliac Disease. Front Immunol (2021) 12:674313. doi: 10.3389/fimmu.2021.674313 34149709 PMC8206552

[28] Moretti S Mrakic-Sposta S Roncoroni L Vezzoli A Dellanoce C Monguzzi E. Oxidative Stress as a Biomarker for Monitoring Treated Celiac Disease. Clin Transl Gastroenterol (2018) 9(6):157. doi: 10.1038/s41424-018-0031-6 29880904 PMC5992147

[29] Chen Y Zhang H Chen Y Jia P Ji S Zhang Y. Resveratrol and Its Derivative Pterostilbene Ameliorate Intestine Injury in Intrauterine Growth-Retarded Weanling Piglets by Modulating Redox Status and Gut Microbiota. J Anim Sci Biotechnol (2021) 12(1):70. doi: 10.1186/s40104-021-00589-9 34108035 PMC8191009

[30] Mayangsari Y Suzuki T. Resveratrol Ameliorates Intestinal Barrier Defects and Inflammation in Colitic Mice and Intestinal Cells. J Agric Food Chem (2018) 66(48):12666–74. doi: 10.1021/acs.jafc.8b04138 30426751

[31] Alharris E Mohammed A Alghetaa H Zhou J Nagarkatti M Nagarkatti P. The Ability of Resveratrol to Attenuate Ovalbumin-Mediated Allergic Asthma Is Associated With Changes in Microbiota Involving the Gut-Lung Axis, Enhanced Barrier Function and Decreased Inflammation in the Lungs. Front Immunol (2022) 13:805770. doi: 10.3389/fimmu.2022.805770 35265071 PMC8898895

[32] Singh AP Singh R Verma SS Rai V Kaschula CH Maiti P. Health Benefits of Resveratrol: Evidence From Clinical Studies. Med Res Rev (2019) 39(5):1851–91. doi: 10.1002/med.21565 30741437

[33] Oliveira A Monteiro VVS Navegantes-Lima KC Reis JF Gomes R Rodrigues DVS. Resveratrol Role in Autoimmune Disease-A Mini-Review. Nutrients (2017) 9(12):1306. doi: 10.3390/nu9121306 PMC5748756 29194364

[34] Zhang C Feng Y Qu S Wei X Zhu H Luo Q. Resveratrol Attenuates Doxorubicin-Induced Cardiomyocyte Apoptosis in Mice Through Sirt1-Mediated Deacetylation of P53. Cardiovasc Res (2011) 90(3):538–45. doi: 10.1093/cvr/cvr022 21278141

[35] Lagouge M Argmann C Gerhart-Hines Z Meziane H Lerin C Daussin F. Resveratrol Improves Mitochondrial Function and Protects Against Metabolic Disease by Activating Sirt1 and Pgc-1alpha. Cell (2006) 127(6):1109–22. doi: 10.1016/j.cell.2006.11.013 17112576

[36] Atreya I Atreya R Neurath MF. Nf-Kappab in Inflammatory Bowel Disease. J Intern Med (2008) 263(6):591–6. doi: 10.1111/j.1365-2796.2008.01953.x 18479258

[37] Nunes S Danesi F Del Rio D Silva P. Resveratrol and Inflammatory Bowel Disease: The Evidence So Far. Nutr Res Rev (2018) 31(1):85–97. doi: 10.1017/S095442241700021X 29191255

[38] Anderson MS Su MA. Aire Expands: New Roles in Immune Tolerance and Beyond. Nat Rev Immunol (2016) 16(4):247–58. doi: 10.1038/nri.2016.9 PMC4831132 26972725

[39] Anderson MS Su MA. Aire and T Cell Development. Curr Opin Immunol (2011) 23(2):198–206. doi: 10.1016/j.coi.2010.11.007 21163636 PMC3073725

[40] Chuprin A Avin A Goldfarb Y Herzig Y Levi B Jacob A. The Deacetylase Sirt1 Is an Essential Regulator of Aire-Mediated Induction of Central Immunological Tolerance. Nat Immunol (2015) 16(7):737–45. doi: 10.1038/ni.3194 26006015

[41] Dikic I Wakatsuki S Walters KJ. Ubiquitin-Binding Domains - From Structures to Functions. Nat Rev Mol Cell Biol (2009) 10(10):659–71. doi: 10.1038/nrm2767 PMC7359374 19773779

[42] Ji S Liu Q Zhang S Chen Q Wang C Zhang W. Fgf15 Activates Hippo Signaling to Suppress Bile Acid Metabolism and Liver Tumorigenesis. Dev Cell (2019) 48(4):460–74.e9. doi: 10.1016/j.devcel.2018.12.021 30745141

[43] Watanabe M Houten SM Mataki C Christoffolete MA Kim BW Sato H. Bile Acids Induce Energy Expenditure by Promoting Intracellular Thyroid Hormone Activation. Nature (2006) 439(7075):484–9. doi: 10.1038/nature04330 16400329

[44] Huang F Zheng X Ma X Jiang R Zhou W Zhou S. Theabrownin From Pu-Erh Tea Attenuates Hypercholesterolemia *Via* Modulation of Gut Microbiota and Bile Acid Metabolism. Nat Commun (2019) 10(1):4971. doi: 10.1038/s41467-019-12896-x 31672964 PMC6823360

[45] Chen M-l Yi L Zhang Y Zhou X Ran L Yang J. Resveratrol Attenuates Trimethylamine-N-Oxide (Tmao)-Induced Atherosclerosis by Regulating Tmao Synthesis and Bile Acid Metabolism *Via* Remodeling of the Gut Microbiota. mBio (2016) 7(2):e02210–5. doi: 10.1128/mBio.02210-15 PMC4817264 27048804

[46] Kim Y-C Byun S Seok S Guo G Xu HE Kemper B. Small Heterodimer Partner and Fibroblast Growth Factor 19 Inhibit Expression of Npc1l1 in Mouse Intestine and Cholesterol Absorption. Gastroenterology (2019) 156(4):1052–65. doi: 10.1053/j.gastro.2018.11.061 PMC6409196 30521806

[47] Kim Y-C Seok S Zhang Y Ma J Kong B Guo G. Intestinal Fgf15/19 Physiologically Repress Hepatic Lipogenesis in the Late Fed-State by Activating Shp and Dnmt3a. Nat Commun (2020) 11(1):5969. doi: 10.1038/s41467-020-19803-9 33235221 PMC7686350

[48] Decara J Rivera P López-Gambero AJ Serrano A Pavón FJ Baixeras E. Peroxisome Proliferator-Activated Receptors: Experimental Targeting for the Treatment of Inflammatory Bowel Diseases. Front Pharmacol (2020) 11:730. doi: 10.3389/fphar.2020.00730 32536865 PMC7266982

[49] Marion-Letellier R Déchelotte P Iacucci M Ghosh S. Dietary Modulation of Peroxisome Proliferator-Activated Receptor Gamma. Gut (2009) 58(4):586–93. doi: 10.1136/gut.2008.162859 19017686

[50] Oeckinghaus A Hayden MS Ghosh S. Crosstalk in Nf-κb Signaling Pathways. Nat Immunol (2011) 12(8):695–708. doi: 10.1038/ni.2065 21772278

[51] Wahli W Michalik L. Ppars at the Crossroads of Lipid Signaling and Inflammation. Trends Endocrinol Metab (2012) 23(7):351–63. doi: 10.1016/j.tem.2012.05.001 22704720

[52] Manoharan I Suryawanshi A Hong Y Ranganathan P Shanmugam A Ahmad S. Homeostatic Pparα Signaling Limits Inflammatory Responses to Commensal Microbiota in the Intestine. J Immunol (2016) 196(11):4739–49. doi: 10.4049/jimmunol.1501489 PMC4875842 27183583

[53] Kanakasabai S Chearwae W Walline CC Iams W Adams SM Bright JJ. Peroxisome Proliferator-Activated Receptor Delta Agonists Inhibit T Helper Type 1 (Th1) and Th17 Responses in Experimental Allergic Encephalomyelitis. Immunology (2010) 130(4):572–88. doi: 10.1111/j.1365-2567.2010.03261.x PMC2913268 20406305

[54] Barish GD Atkins AR Downes M Olson P Chong L-W Nelson M. Ppardelta Regulates Multiple Proinflammatory Pathways to Suppress Atherosclerosis. Proc Natl Acad Sci USA (2008) 105(11):4271–6. doi: 10.1073/pnas.0711875105 PMC2393796 18337509

[55] Neels JG Grimaldi PA. Physiological Functions of Peroxisome Proliferator-Activated Receptor β. Physiol Rev (2014) 94(3):795–858. doi: 10.1152/physrev.00027.2013 24987006

[56] Cantó C Gerhart-Hines Z Feige JN Lagouge M Noriega L Milne JC. Ampk Regulates Energy Expenditure by Modulating Nad+ Metabolism and Sirt1 Activity. Nature (2009) 458(7241):1056–60. doi: 10.1038/nature07813 PMC3616311 19262508

[57] Ma EH Poffenberger MC Wong AHT Jones RG. The Role of Ampk in T Cell Metabolism and Function. Curr Opin Immunol (2017) 46:45–52. doi: 10.1016/j.coi.2017.04.004 28460345

[58] Blagih J Coulombe F Vincent EE Dupuy F Galicia-Vázquez G Yurchenko E. The Energy Sensor Ampk Regulates T Cell Metabolic Adaptation and Effector Responses *In Vivo* . Immunity (2015) 42(1):41–54. doi: 10.1016/j.immuni.2014.12.030 25607458

[59] Alghetaa H Mohammed A Zhou J Singh N Nagarkatti M Nagarkatti P. Resveratrol-Mediated Attenuation of Superantigen-Driven Acute Respiratory Distress Syndrome Is Mediated by Microbiota in the Lungs and Gut. Pharmacol Res (2021) 167:105548. doi: 10.1016/j.phrs.2021.105548 33722710 PMC10116750

[60] O’Neill LAJ Hardie DG. Metabolism of Inflammation Limited by Ampk and Pseudo-Starvation. Nature (2013) 493(7432):346–55. doi: 10.1038/nature11862 23325217

[61] Salt IP Palmer TM. Exploiting the Anti-Inflammatory Effects of Amp-Activated Protein Kinase Activation. Expert Opin Investig Drugs (2012) 21(8):1155–67. doi: 10.1517/13543784.2012.696609 22694351

[62] Caminero A McCarville JL Zevallos VF Pigrau M Yu XB Jury J. Lactobacilli Degrade Wheat Amylase Trypsin Inhibitors to Reduce Intestinal Dysfunction Induced by Immunogenic Wheat Proteins. Gastroenterology (2019) 156(8):2266–80. doi: 10.1053/j.gastro.2019.02.028 30802444

[63] Pineda CT Ramanathan S Fon Tacer K Weon JL Potts MB Ou Y-H. Degradation of Ampk by a Cancer-Specific Ubiquitin Ligase. Cell (2015) 160(4):715–28. doi: 10.1016/j.cell.2015.01.034 PMC5629913 25679763

[64] Zhou W Cao Q Peng Y Zhang Q-J Castrillon DH DePinho RA. Foxo4 Inhibits Nf-Kappab and Protects Mice Against Colonic Injury and Inflammation. Gastroenterology (2009) 137(4):1403–14. doi: 10.1053/j.gastro.2009.06.049 PMC2764529 19560465

[65] Zhao Y Hu X Liu Y Dong S Wen Z He W. Ros Signaling Under Metabolic Stress: Cross-Talk Between Ampk and Akt Pathway. Mol Cancer (2017) 16(1):79. doi: 10.1186/s12943-017-0648-1 28407774 PMC5390360

