# Supplementary figures and images for: The Nutritional Intervention of Resveratrol Can Effectively Alleviate the Intestinal Inflammation Associated With Celiac Disease Induced by Wheat Gluten
Source: Front Immunol. 2022 Apr 5;13:878186. doi: 10.3389/fimmu.2022.878186 (PMC9017684; doi:10.3389/fimmu.2022.878186)

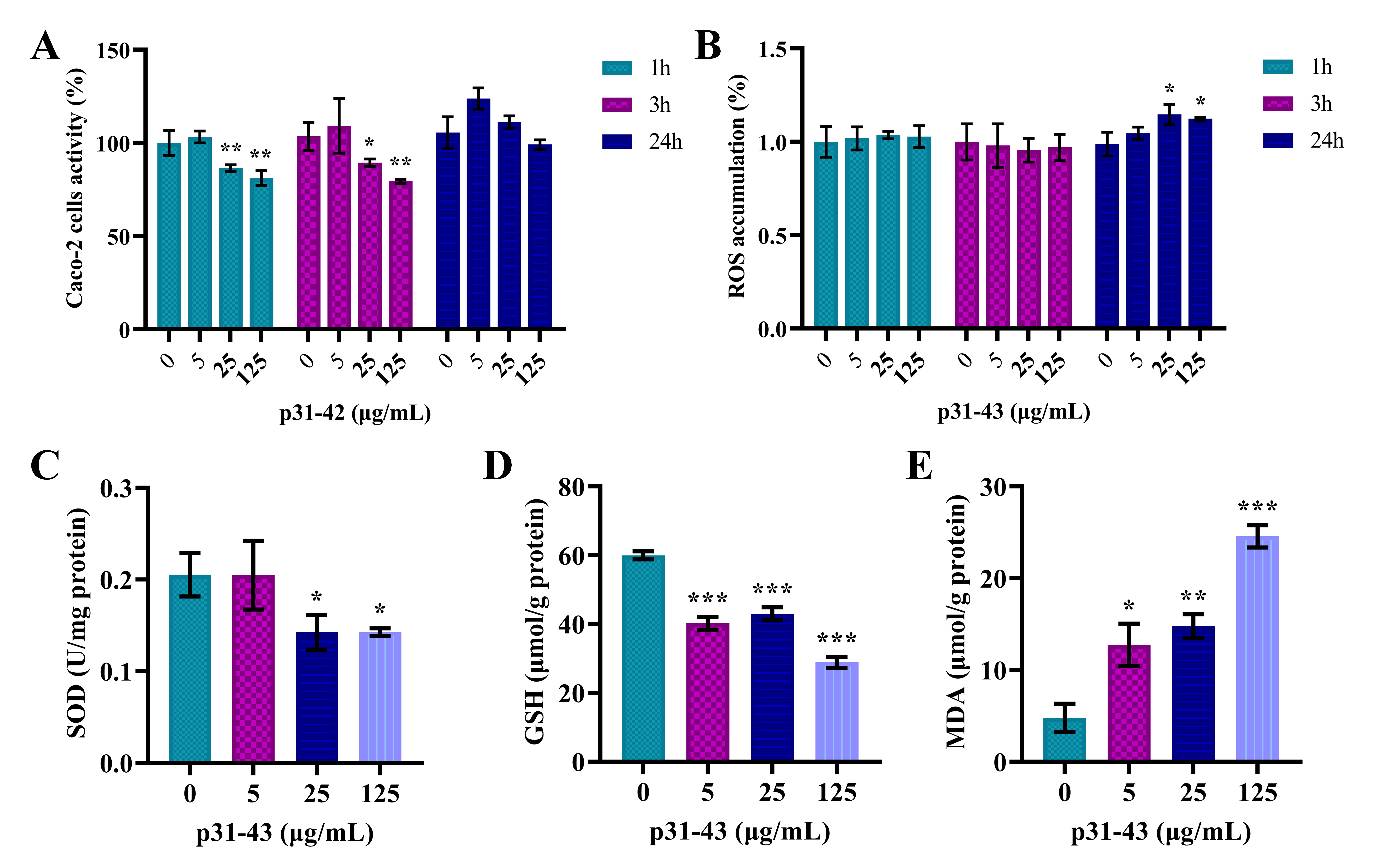

Supplement: Supplementary Figure 1 — Gluten protein-derived peptide p31-43 induced Caco-2 cells to construct a celiac oxidative stress model. (A) Changes of caco-2 cells activity. (B) The accumulation of intracellular ROS. Changes in SOD enzyme activity (C), GSH content (D) and MDA content (E) in Caco-2 cells after 24 h of P31-43 peptide stimulation. * indicated statistically significant differences when compared to the PBS group. *P < 0.05; **P < 0.01; ***P < 0.001. [file Image_1.tif]

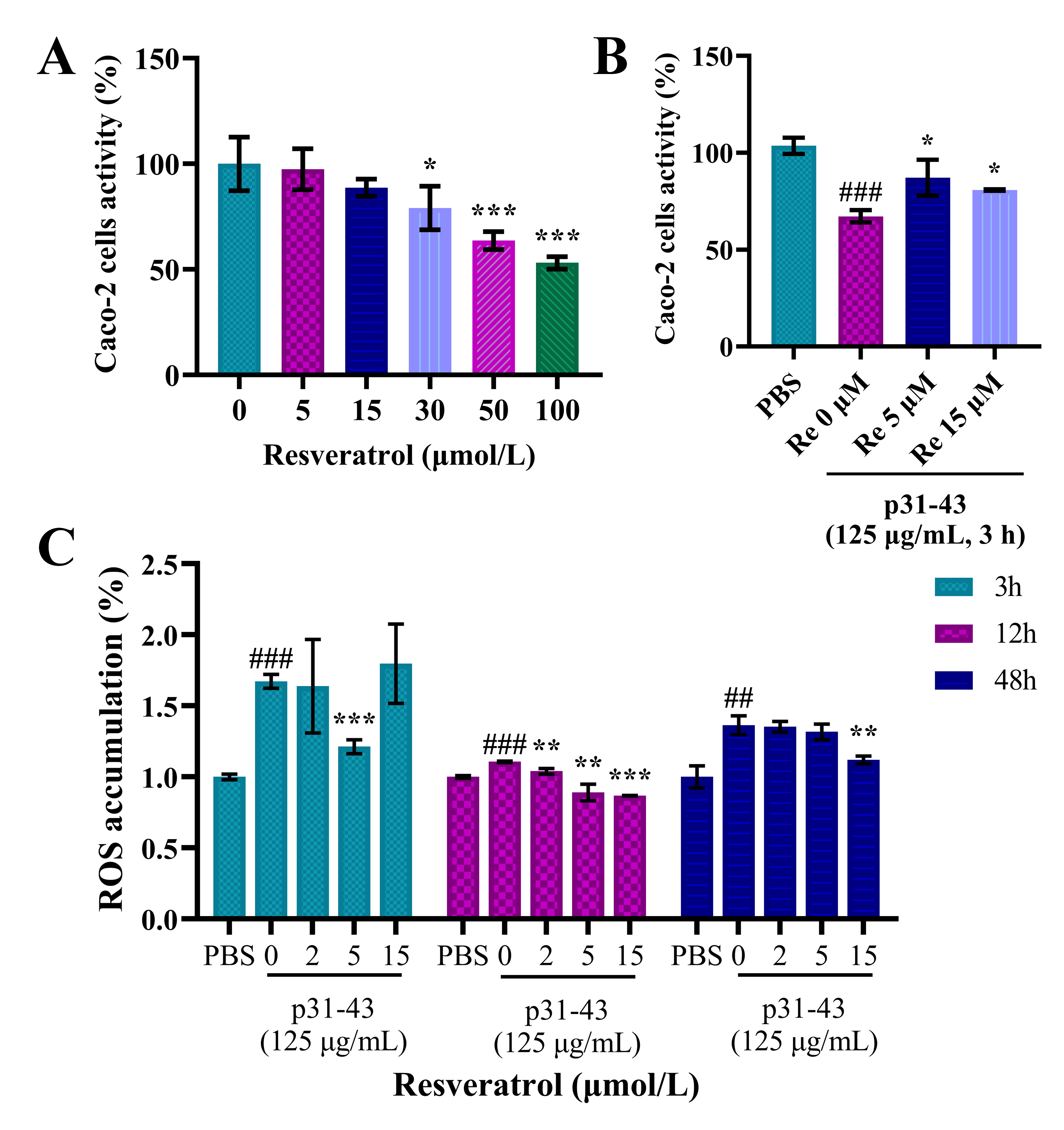

Supplement: Supplementary Figure 2 — Determination of resveratrol concentration and pretreatment time. (A) Safety concentrations of resveratrol in Caco-2 cells. * indicated statistically significant differences when compared to the PBS group. *P < 0.05; ***P < 0.001. (B) Effects of resveratrol on cell viability loss induced by p31-43. (C) Regulation of resveratrol on intracellular ROS accumulation in Caco-2 cells. # indicated statistically significant differences compared to the PBS group. ##P < 0.01; ###P < 0.001. * indicated statistically significant differences when compared to the control group. *P < 0.05; **P < 0.01; ***P < 0.001. [file Image_2.tif]

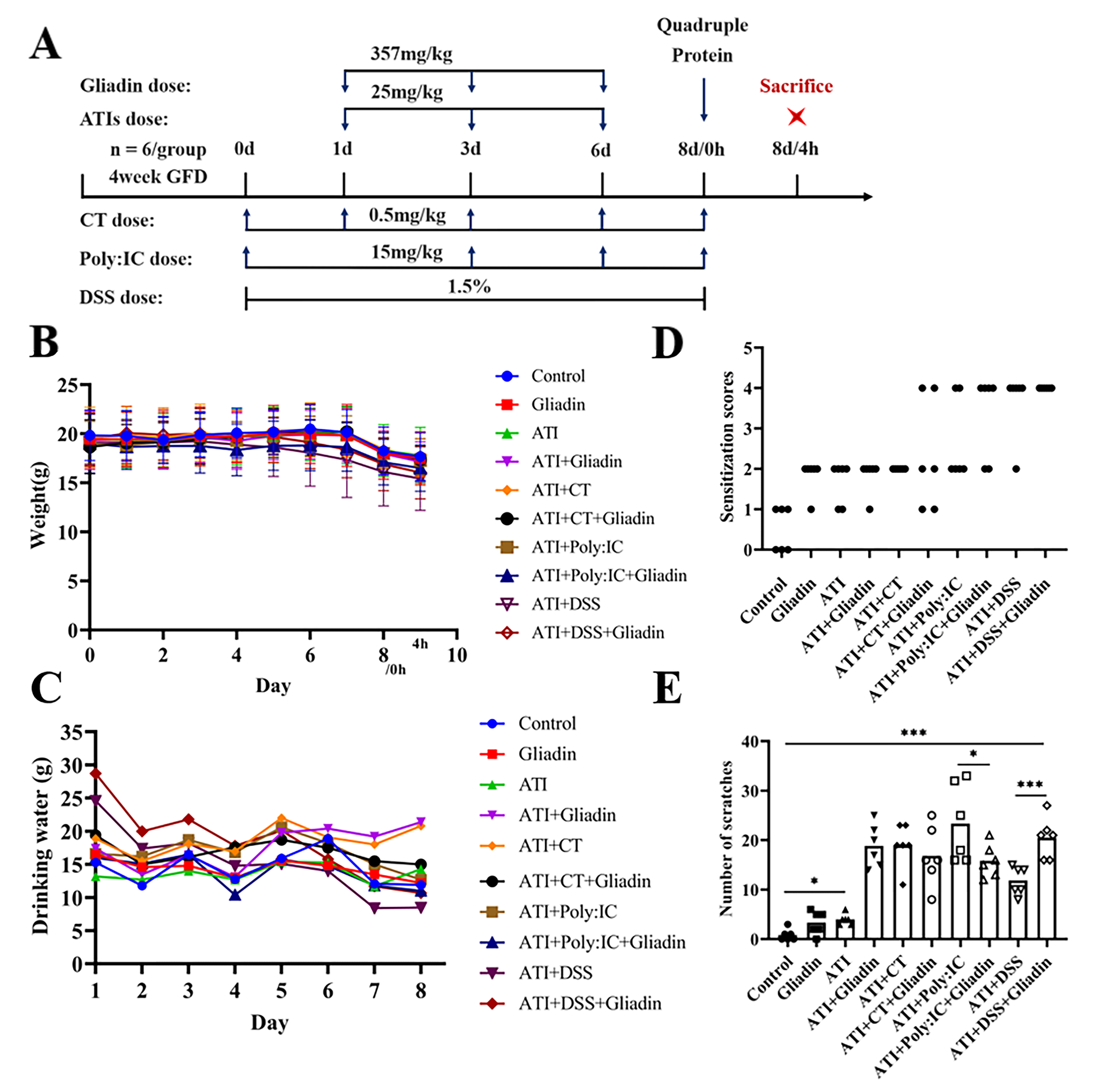

Supplement: Supplementary Figure 3 — Construction of a mouse model for celiac disease. (A) Experimental protocol for model construction. (B) Weight of mice. (C) Water consumption of mice. (D) Clinical symptoms of mice. (E) Number of scratches in mice. *P < 0.05; **P < 0.01; ***P < 0.001. Symptom scoring: (0) No symptoms; (1) Scratching nose and mouth; (2) Swelling around the eyes and mouth, diarrhea, reduced activity or walking in place, higher breathing rate; (3) Shortness of breath, wheezing, blue rash around the mouth and tail; (4) Loss of consciousness, tremors or cramps, blood in stool; (5) Death by shock. [file Image_3.tif]

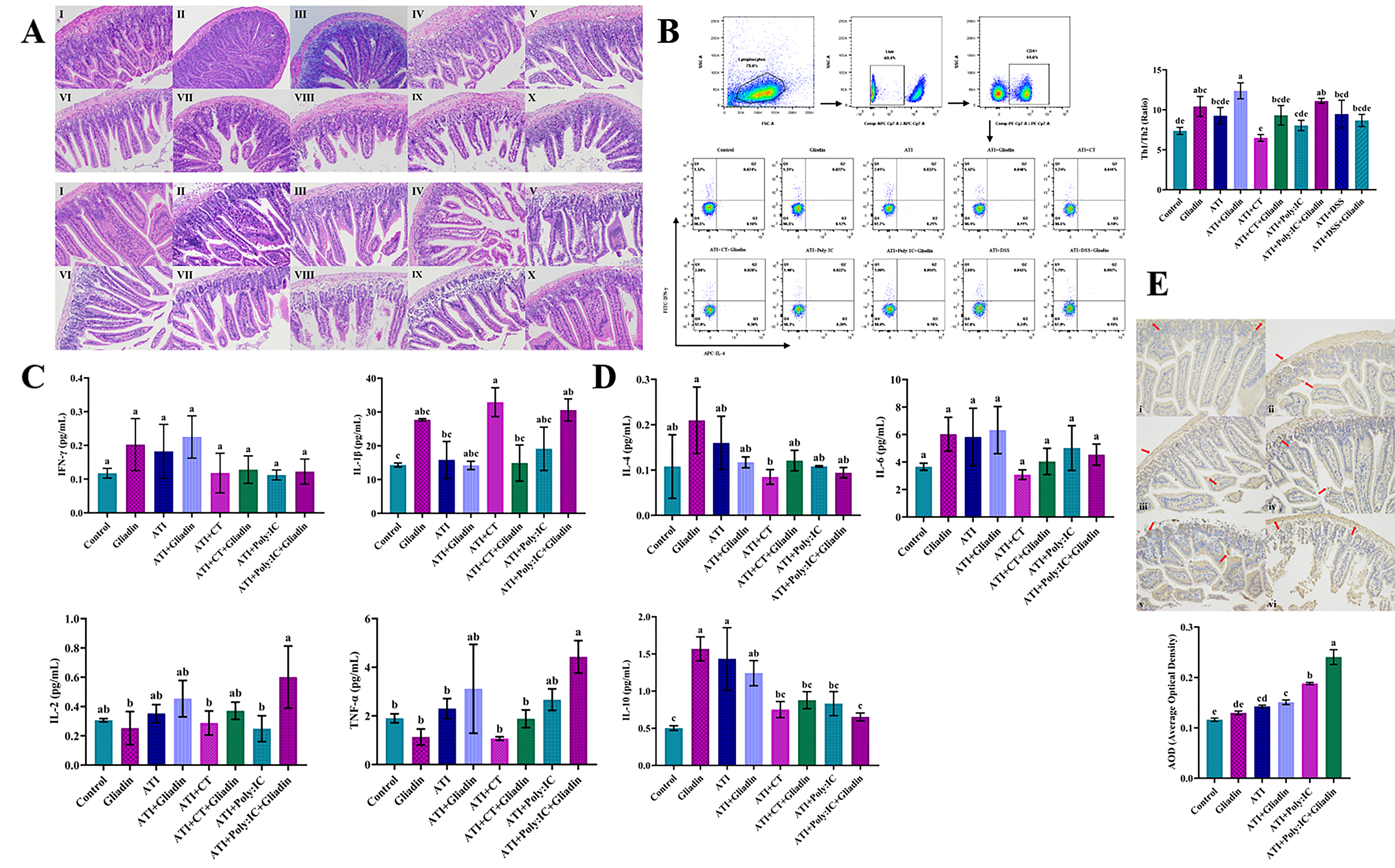

Supplement: Supplementary Figure 4 — Characteristic indicators of the mouse model for celiac disease. (A) Intestine histological sections of ileum (above) and jejunum (below) stained with hematoxylin and eosin stain from mice. (B) The ratios of Th1/Th2 cells in mesenteric lymph nodes of mouse. (C) The expression level of Th1 related inflammatory factors (IFN-γ, IL-1β, IL-2, TNF-α), (D) Th2 related inflammatory factors (IL-4, IL-6) and IL-10 in the lysate of jejunum tissue. (E) Immunohistochemistry analysis of tissue transglutaminase in jejunum tissue sections. Different alphabets of a-e indicated statistically significant differences (p < 0.05). (A) were the fields of view at 200x lens. I: Control group; II: Gliadin group; III;: ATI group; IV: ATI+Gliadin group; V: ATI+CT group; VI: ATI+CT+Gliadin group; VII: ATI+Poly : IC group; VIII: ATI+Poly: IC+Gliadin group; IX: ATI+DSS group; X: ATI+DSS+Gliadin group. Figure E were the fields of view at 200x lens. i: Control group; ii: Gliadin group; iii: ATI group; iv: ATI+Gliadin group; v: ATI+Poly : IC group; vi: ATI+Poly : IC+Gliadin group. [file Image_4.tif]

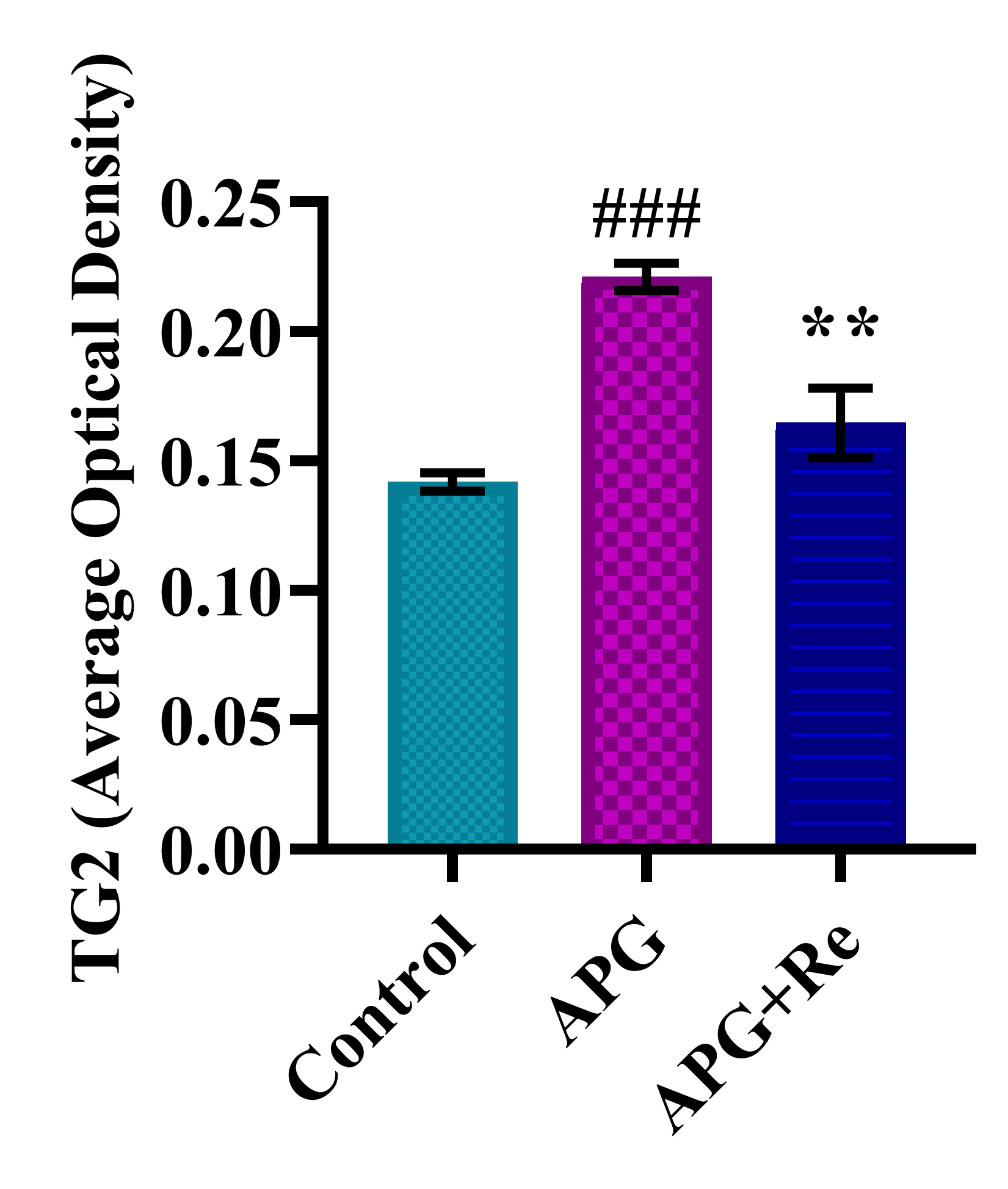

Supplement: Supplementary Figure 5 — Average optical density analysis of tissue transglutaminase in jejunum tissue sections. [file Image_5.tif]
