# Supplementary material for: The Nutritional Intervention of Resveratrol Can Effectively Alleviate the Intestinal Inflammation Associated With Celiac Disease Induced by Wheat Gluten
Source: Front Immunol. 2022 Apr 5;13:878186. doi: 10.3389/fimmu.2022.878186 (PMC9017684; doi:10.3389/fimmu.2022.878186)
Supplement: Supplementary file 6 [file DataSheet_1.docx]

**Supplemental Materials and Methods**

**Reagents**

Enhanced BCA Protein Assay Kit, Total Superoxide Dismutase Assay Kit with WST-8, Lipid Peroxidation MDA Assay Kit, Nitric Oxide Assay Kit and Cell Counting Kit-8 were purchased from Beyotime Biotechnology (Beyotime, Shanghai, China). Reactive oxygen species (ROS) assay Kit and Reduced glutathione (GSH) assay kit were acquired from Njjcbio (Nanjing Jiancheng, Jiangsu, China). Polyinosinic: polycytidylic acid (Poly : IC) and dextran sulfate sodium salt (DSS, MW: 40,000 Da) were purchased from Sigma-Aldrich (Sigma, Missouri, USA). Cholera Toxin (CT, B subunit) was acquired from Absin (Shanghai, China).

**Establishment of Cell Model**

The frozen Caco-2 cells were placed in a 37°C thermostatic water bath and gently shaken to lyse them rapidly. The cells were transferred to centrifuge tubes after 2 min, followed by the addition of 5 mL complete medium and centrifuged at 100 g for 5 min. After discarding the supernatant, 3 mL complete medium was added to the sediment. The cells were gently dispersed with a pasteur pipette and transferred to the culture flask, which was replenished to 10-12 mL with complete medium. The cell culture flasks were incubated at 37°C in an incubator with 90% relative air humidity and 5% CO2.

When the cell fusion rate reached 95%, the cells were digested with 2 mL EDTA-trypsin for 3 min and observed under the microscope. After the cells were digested into an ellipsoidal shape, the digestion was terminated by adding 4 mL complete medium, and the cells were gently blown out into a single-cell suspension with an electric pipette. The suspension was centrifuged at 100 g for 3 min, followed by discarding the supernatant and adding complete medium. The cells were passaged at a ratio of 1:3. After the cells grew to logarithmic phase, the operation of passaging was performed. When starting the experiment, the cells were diluted to 2×10^5^ cells/mL with complete medium.

We set different concentrations of peptide p31-43 of gliadin to stimulate Caco-2 cells for 1 h, 3 h and 24 h, respectively, and determined the optimal cell model based on the accumulation of ROS, activity of SOD enzyme, content of GSH and MDA in Caco-2 cells. The working parameter of the celiac disease oxidative stress model was 125 μg/mL p31-43 peptide to stimulate Caco-2 cells for 24 h. The working parameter of model for detecting changes in cell viability was 125 μg/mL p31-43 peptides to stimulate Caco-2 cells for 3 h.

**Cell Activity**

Caco-2 cells activity was determined following the manufacturer’s instructions of the Cell Counting Kit-8.

**Determination of Oxidative Stress Indexes**

Caco-2 cells were assayed for intracellular ROS content by the DCFH-DA probe. Caco-2 cells were lysed to determine the protein concentration, with subsequent determination of GSH content, MDA content and SOD enzyme activity using the kit. All the procedure was followed on the protocol of the kit (code: ROS Njjcbio E00411, NO Beyotime S0021S, GSH Njjcbio A00621, MDA Beyotime S0131M and SOD Beyotime S0101M).

**Establishment of Mouse Model**

The specific pathogen free (SPF) C57BL/6N mice (20 ± 2.0 g) of 6 weeks were purchased from Beijing Vital River Laboratory Animal Technology Co., Ltd. (Beijing, China) (License No. SCXK [Beijing] 2016-0006) and all the mice used in this study were took cared in accordance with the Guidelines for Animal Experimentation of the Laboratory Animal Research center for Science and Technology of Jiangxi University of Traditional Chinese Medicine (Nanchang, China) (License No. SYXK[Jiangxi] 2017-0004). All experimental procedures were examined and approved by the Experimental Animal Ethics Committee of Jiangxi University of Traditional Chinese Medicine (No. JZSYDWLL-20200515; date of approval: 19 May 2020). The mice were fed a gluten-free diet (based on AIN-93 standard, Trophic Animal Feed High-Tech Co., Ltd, China) with a defined carbohydrate content and casein as main protein source and continuously propagated for two generations at least, and the second generation and above of the female mice (6 weeks old) were used as experiment animals to circumvent gluten exposure. The room temperature and humidity were maintained under conditions of 21 °C–25 °C and 55%–65%, respectively, with 12 h dark & light cycle.

We referred to the experiments of Zevallos (18) and Caminero (60) to establish a mouse model of celiac disease by using cholera toxin (CT), polyinosinic:polycytidylic acid (Poly:IC) and dextran sodium sulfate (DSS). Ten groups were established, named Control group, Gliadin group, ATI group, ATI+Gliadin group, ATI+CT group, ATI+CT+Gliadin group, ATI+Poly:IC group, ATI+Poly:IC+Gliadin group, ATI+DSS group and ATI+DSS+Gliadin group. Supplementary Figure 3A showed the time diagram of the experimental protocol, and the body weight, water intake and symptom scores of mice were recorded during the experiment. The mice were stopped from food supply 12 h before slaughter. Mice were stimulated with quadruple protein on day 8 and were executed 4 hours later. The doses were ATI 25 mg/kg, Gliadin 357 mg/kg (10 mg/0.028 kg), CT 0.5 mg/kg, Poly:IC 15 mg/kg and DSS 1.5%. When Poly:IC was injected intraperitoneally, saline was injected intraperitoneally in all other groups. The DSS solution was reconfigured every two days, while the water consumption was recorded.

**Histology and Immunohistochemistry**

The intestinal tissues of mice were fixed by fixed liquid and stained with hematoxylin-eosin. After it was dehydrated and sealed with neutral gum, the sections were observed by an optical microscope, followed by recording the images of the tissue scanning (upright optical microscope Nikon Eclipse E100 and imagine system Nikon DS-U3, Nikon Co., Japan). The paraffin-dehydrated sections were subjected to antigen repair, blocking, incubation of primary and secondary antibodies, color development and image acquisition. The average optical density value was used to calculate the proportion of positive areas of TG2 on immunohistochemical sections compared to intestinal tissue, which indirectly reflects the amount of TG2 in the intestine. These stained sections were visualized and photographed under optical microscope (200× magnification). The IHC data were quantified by Image J software. Different parts of the same tissue would be analyzed.

**Detection of Th1 and Th2**

The mesenteric lymph nodes (MLN) of mice were ground and re-suspended to 5*10^6 cell/mL which were subsequently stimulated *in vitro* for 16 h. The cell suspensions of MLN were washed with azide-free and serum/protein-free PBS to perform live-death staining by Fixable Viability Dye 780 (Thermo Fisher scientific, Massachusetts, USA). The cells of MLN were washed using a treatment solution containing fetal bovine serum and stained for CD4 cells (PE/Cy7 anti-mouse CD4 Antibody, BD Biosciences, New Jersey, USA). Moreover, the cells were fixed and stained for cell permeabilization (APC anti-mouse IL-4 and FITC anti-mouse IFN-γ, respectively, BD Biosciences, New Jersey, USA). Then, the cells were re-suspended after incubation in the dark, followed by analyzing detection of Th1/Th2 using flow cytometry (BD FACSVerse, USA).

**Cytokine Analysis**

The jejunal tissue were lysed under lysis solution, followed by centrifugation at 10,000 g at 4℃. After determining the concentration, the supernatant was assayed for cytokines (included IL-1β, TNF-α, IFN-γ, IL-2, IL-4, IL-6 and IL-10) using the Meso Scale Discovery kit (Meso Scale Diagnostics, Rockville, MD, USA) followed the manufacturer’s instructions. Cytokine levels were determined using a MESOTM QuickPlex SQ 120 (Meso Scale Diagnostics, Rockville, MD, USA).

**Statistical Analysis**

The IHC toolbox plug-in of Image J software was used to analyze the immunohistochemistry data and the Average Optical Density (AOD) was used to calculate the positive area of the immunohistochemistry section. The data of flow cytometry were analyzed by BD FACSuite software and FlowJo V10 software. Results were analyzed by One-way ANOVA in IBM SPSS Statistics 24 software, where mean differences were analyzed using Tukey's HSD test and independent samples t-test, and probability values of p < 0.05 were considered significantly different. Graphs were drawn using GraphPad Prism 8, Origin 2019, BioRender and PowerPoint 2016.

The Sequence of Primers

| Gene | Primer Sequence |
| --- | --- |
| TrxR1 | F:CCACTGGTGAAAGACCACGTT |
|  | R:AGGAGAAAAGATCATCACTGCTGAT |
| HO-1 | F:TCCGATGGGTCCTTACACTC |
|  | R:AAGGAAGCCAGCCAAGAGA |
| NQO1 | F:CGCAGACCTTGTCATATTCCAG |
|  | R:TCCTATGAACACTCGCTCAAACC |
| Nrf2 | F:AGTGGATCTGCCAACTACTC |
|  | R:CATCTACAAACGGGAATGTCTG |
| TG2 | F:TGCTGTGGAGGAGGGTGACT |
|  | R:ACCAGGCGTTGAAGAGCAAA |
| iNOS | F:TCCGAGGCAAACAGCACATTCA |
|  | R:GGGTTGGGGGTGTGGTGATGT |
| COX-2 | F:GCCTTCTCTAACCACTCC |
|  | R:CTGATGCGTGAAGTGC |
| β-actin | F:AGCGGGAAATCGTGCGTGAC |
|  | R:TCCATGCCCAGGAAGGAAGG |
| 18S rRNA | F:GGCTCATTAAATCAGTTATCCTTCCT |
|  | R:GTATTAGCTCTAGAATTACCACAGTTATCCA |
| PI3K 110α | F:CCACGACCATCATCAGGTCAA |
|  | R:CCTCACGGAGGCATTCTAAAGT |
| RHOA | F:AGTTTCTTCCGGATGGCAG |
|  | R:CGGTCTGGTCTTCAGCTACC |
| MLCK | F:CACCGTCCATGAAAAGAAGAGTAG |
|  | R:GAGAGGCCCTGCAGGAAGATGG |
| Sirt1 | F:TAGCCTTGTCAGATAAGGAAGGA |
|  | R:ACAGCTTCACAGTCAACTTTGT |
| Sirt2 | F:TGCGGAACTTATTCTCCCAGA |
|  | R:GAGAGCGAAAGTCGGGGAT |
| Sirt3 | F:CCCCAAGCCCTTTTTCACTTT |
|  | R:CGACACTCTCTCAAGCCCA |
| Sirt4 | F:AGCCTCCATTGGGTTATTTGTG |
|  | R:TCTGGTATCCCCGATTCGGT |
| Sirt5 | F:GCCATAGCCGAGTGTGAGAC |
|  | R:CAACTCCACAAGAGGTACATCG |
| Sirt6 | F:CCCACGGAGTCTGGACCAT |
|  | R:CTCTGCCAGTTTGTCCCTG |
